# New perspectives on butyrate assimilation in *Rhodospirillum rubrum* S1H under photoheterotrophic conditions

**DOI:** 10.1186/s12866-020-01814-7

**Published:** 2020-05-20

**Authors:** Quentin De Meur, Adam Deutschbauer, Matthias Koch, Guillaume Bayon-Vicente, Paloma Cabecas Segura, Ruddy Wattiez, Baptiste Leroy

**Affiliations:** 1 Laboratory of Proteomics and Microbiology, Research Institute for Biosciences, University of Mons, Avenue du champs de Mars, 6 (Pentagone 3B), 7000 Mons, Belgium; 2 Environmental Genomics and Systems Biology Division, Lawrence Berkeley National Laboratory, Berkeley, California USA

**Keywords:** Photoheterotrophy, Butyrate assimilation, Ethylmalonyl-CoA, Polyhydroxybutyrate, Anaplerosis

## Abstract

**Background:**

The great metabolic versatility of the purple non-sulfur bacteria is of particular interest in green technology. *Rhodospirillum rubrum* S1H is an α-proteobacterium that is capable of photoheterotrophic assimilation of volatile fatty acids (VFAs). Butyrate is one of the most abundant VFAs produced during fermentative biodegradation of crude organic wastes in various applications. While there is a growing understanding of the photoassimilation of acetate, another abundantly produced VFA, the mechanisms involved in the photoheterotrophic metabolism of butyrate remain poorly studied.

**Results:**

In this work, we used proteomic and functional genomic analyses to determine potential metabolic pathways involved in the photoassimilation of butyrate. We propose that a fraction of butyrate is converted to acetyl-CoA, a reaction shared with polyhydroxybutyrate metabolism, while the other fraction supplies the ethylmalonyl-CoA (EMC) pathway used as an anaplerotic pathway to replenish the TCA cycle. Surprisingly, we also highlighted a potential assimilation pathway, through isoleucine synthesis and degradation, allowing the conversion of acetyl-CoA to propionyl-CoA. We tentatively named this pathway the methylbutanoyl-CoA pathway (MBC). An increase in isoleucine abundance was observed during the early growth phase under butyrate condition. Nevertheless, while the EMC and MBC pathways appeared to be concomitantly used, a genome-wide mutant fitness assay highlighted the EMC pathway as the only pathway strictly required for the assimilation of butyrate.

**Conclusion:**

Photoheterotrophic growth of *Rs. rubrum* with butyrate as sole carbon source requires a functional EMC pathway. In addition, a new assimilation pathway involving isoleucine synthesis and degradation, named the methylbutanoyl-CoA (MBC) pathway, could also be involved in the assimilation of this volatile fatty acid by *Rs. rubrum*.

## Background

Purple non-sulfur bacteria (PNSB) are known for their remarkable metabolic versatility. They represent a promising resource for biotechnology such as biofuel and biodegradable plastic production, wastewater treatment, or biofertilization. *Rhodospirillum rubrum* S1H (*Rs. rubrum*) is an α-proteobacterium which has been chosen by the European Space Agency (ESA) to recycle the volatile fatty acids (VFAs) produced in the MELiSSA (Micro-Ecological Life Support System Alternative) loop, envisaged to autonomously sustain the metabolic needs of a crew for a long-term space mission [1, 2]. In anoxygenic conditions, *Rs. rubrum* is known to grow photoheterotrophically, using light as an energy source and VFAs as carbon and electron sources. VFAs are common byproducts from fermentative processes. They are produced in the first compartment of the MELiSSA loop where raw biomass is liquefied and subjected to fermentation. Similar to acetate, butyrate is one of the most abundant VFAs, and is usually produced during acidogenic digestion [3].

Despite a growing interest in biotechnology, the assimilation metabolism of butyrate under photoheterotrophic conditions remains relatively poorly described. Under catabolic conditions in *Desulfosarcina cetonica*, butyrate can be used as carbon and energy sources. Butyrate is completely oxidized into CO_2_ through the formation of acetyl-CoA which is further oxidized through the acetyl-CoA/carbon monoxide dehydrogenase pathway [4]. In photoheterotrophic conditions with light as the energy source, the assimilated organic carbon compounds are used to supply the biosynthesis of anabolic precursors. These precursors are mainly intermediates of the citric acid cycle and must be continuously replenished through anaplerotic pathways. Sixty years ago, the glyoxylate cycle was the first anaplerotic pathway that had been proposed for the net synthesis of malate from two molecules of acetyl-CoA [5]. Nevertheless, several organisms including *Rs. rubrum* lack the key enzyme of this pathway, the isocitrate lyase (Icl). Among the alternative pathways recently proposed for Icl-negative organisms, the ethylmalonyl-CoA pathway was demonstrated to be a key acetate assimilation route in *Rhodobacter sphaeroides* [6], *Methylobacterium extorquens* [7–9] and *Rs. rubrum* [10].

This study aimed to offer a better understanding of the photoassimilation of butyrate in *Rs. rubrum* under photoheterotrophic conditions, using growth analysis, proteomics, targeted mutagenesis, and genome-wide mutant fitness profiling. We highlighted two main assimilation pathways for butyrate. First, the expected conversion into acetyl-CoA, and the anaplerotic ethylmalonyl-CoA pathway was detected. Second, we also highlighted a potential and unexpected new assimilation pathway involving enzymes related to the biosynthesis and degradation of branched-chain amino acids.

## Results

### Growth of *Rs. rubrum* S1H under photoheterotrophic conditions with butyrate as carbon source

**Fig. 1:**
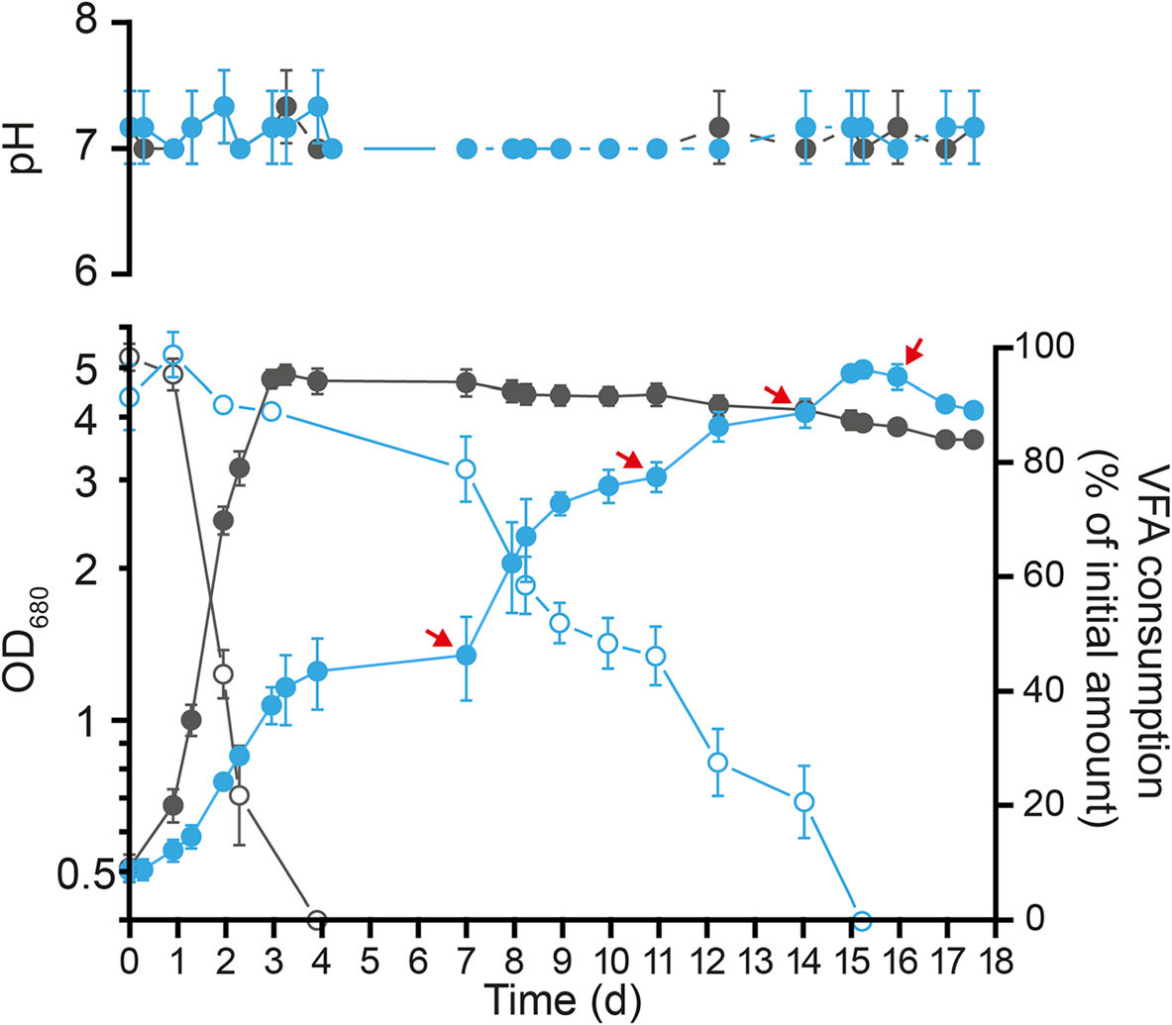
Growth phenotype characterization of *Rs. rubrum* S1H under photoheterotrophic conditions with butyrate as unique carbon source. Monitoring of the growth (filled markers) and VFA consumption (opened markers) of *Rs. rubrum* S1H under light anaerobic conditions using succinate (gray; *n* = 3) or butyrate (blue circles; n = 3) as unique carbon sources. Red arrows represent for bicarbonate supplementation (70 μmoles of NaHCO_3_, 3 ± 0.5 mM final). The growth was monitored by measuring the OD_680nm_. The VFA abundances were measured in culture supernatants using HPLC and refractometric detection

To obtain unbiased proteomic data, it is crucial to harvest cells grown under different conditions in similar growth phases. Thus, we first characterized the growth behavior of *Rs. rubrum* S1H in photoheterotrophic conditions with butyrate or succinate as the carbon sources. Compared to succinate, we observed a drastically lower biomass production in the presence of butyrate and 3 mM bicarbonate with stationary phase appearing at an OD_680nm_ of 1.3 while the OD_680nm_ reached almost 5 in the presence of succinate (Fig. 1). Monitoring of the butyrate concentration in the culture medium highlighted the incomplete consumption of butyrate. The full consumption of butyrate and the production of equivalent biomass compared to succinate were possible only by supplementing the medium with additional bicarbonate (Fig. 1, red arrows). Indeed, we observed three successive growth arrests in the growth curve that were concomitant with halted butyrate assimilation and that could all be released by the addition of extra bicarbonate. Notably, the fourth addition of bicarbonate, when butyrate was completely exhausted from the medium, did not trigger additional growth, confirming that bicarbonate is involved in butyrate assimilation and not in autotrophic growth. To rule out an effect of the addition of bicarbonate on the pH of the culture, the pH has been monitored along the culture and no modification were observed (Fig. 1).

### Proteomic analysis of butyrate photoassimilation

**Fig. 2:**
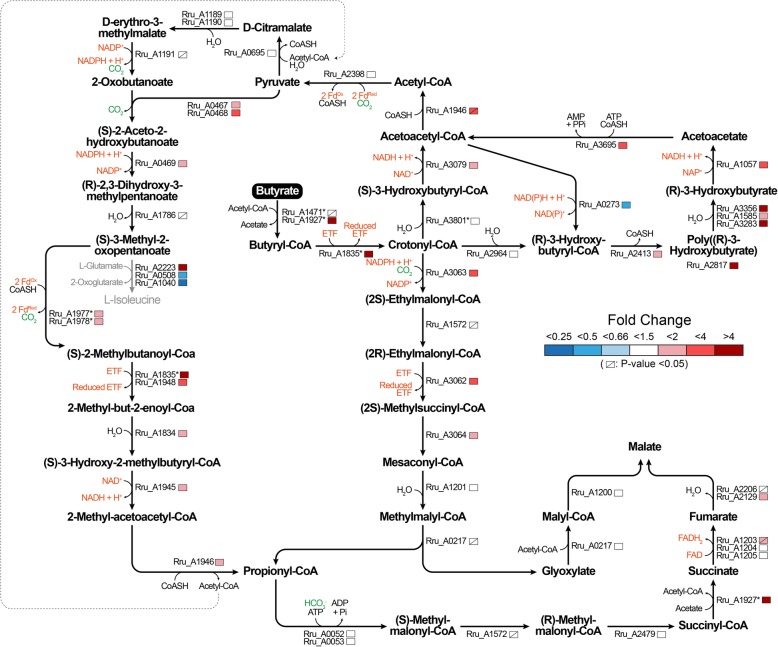
Schematic representation of the metabolic pathways highlighted based on proteomic data. The colored markers indicate the fold change, ranging from blue (downregulation under the butyrate growth conditions compared to the control succinate growth conditions) to red (upregulation). Strikethrough markers represent non-significant fold changes (*P*-value > 0.05). Enzymes marked with a star have yet to be confirmed due to either possible multiple assignations in proposed pathways or to uncertain enzymatic activity. ETF stands for electron transfer flavoproteins. Fd stands for ferredoxins

To gain better understanding of the metabolic pathways responsible for butyrate photoassimilation, we compared the proteome of the biomass obtained under butyrate and succinate growth conditions. To avoid bias related to different illumination perceived at the cellular level, the biomass was harvested at equivalent cell densities (OD_680nm_ of 1.5–2.0). Five biological replicates (independent clonal cultures) were considered. With 1752 proteins identified (at a global false discovery rate below 1%) and quantified, these proteomic data covered approximately half of the theoretical proteome of *Rs. rubrum* S1 (ATCC1170, 3836 entries in UniProt). The complete dataset is available in the supporting material (Table S1) and the data discussed below are listed in the Table 1. Among the 353 proteins quantified with more than one peptide and showing a significant and biologically relevant fold change (*P*-value< 0.05; fold change > 1.5 or < 0.66), 162 and 191 proteins were quantified with higher or lower abundances, respectively, under butyrate growth condition compared to succinate growth condition. Our proteomic data lead us to propose that multiple metabolic pathways are involved in the photoassimilation of butyrate. These pathways are depicted in Fig. 2 and the interpretation of the proteomic data is detailed in the discussion section.

**Table 1:** Differential protein expressions in *Rhodospirillum rubrum* S1H

Locus	Protein	FC^**a**^ B/S	***P***^**b**^	pept ^**c**^	FC^**a**^ T1/T0	***P***^**b**^	FC^**a**^ T2/T1	***P***^**b**^	FC^**a**^ T3/T2	***P***^**b**^	FC^**a**^ T4/T3	***P***^**b**^	pept ^**c**^
Rru_A1471	Butyryl-CoA:acetate CoA transferase	1.5	7e-01	1	n.a.	n.a.	n.a.	n.a.	n.a.	n.a.	n.a.	n.a.	n.a.
Rru_A1927	Acetyl-CoA hydrolase	4.1	2e-10	6	1.0	5e-01	1.0	9e-01	1.0	1e+ 00	0.5	3e-08	6
Rru_A3801	Short chain enoyl-CoA hydratase	1.2	1e-02	6	1.0	1e+ 00	1.1	7e-01	1.0	8e-01	1.0	5e-02	6
Rru_A3079	3-hydroxyacyl-CoA dehydrogenase	1.9	5e-08	5	1.0	3e-01	0.9	2e-02	1.3	4e-05	0.9	2e-01	6
Rru_A1946	Acetoacetyl-CoA thiolase	3.7^d^	2e-02	2	0.8	3e-02	0.8	1e-02	1.2	7e-03	0.9	2e-01	3
Rru_A0273	Acetoacetyl-CoA reductase	0.3	4e-07	5	0.8	7e-05	0.9	2e-02	1.2	8e-05	1.4	1e-05	6
Rru_A2964	(R)-specific enoyl-CoA hydratase	0.9	5e-01	6	0.8	4e-01	1.2	9e-01	0.7	7e-01	1.7	6e-03	5
Rru_A2413	Poly(R)-hydroxyalkanoic acid synthase	1.7	1e-04	5	1.0	3e-01	1.1	5e-02	1.0	3e-01	1.5	4e-06	6
Rru_A2817	Phasin	49.8	5e-06	6	0.4	2e-03	1.1	8e-01	0.7	2e-02	0.2	1e-04	6
Rru_A3283	Activator of polymer mobilization	9.7	6e-06	6	0.9	7e-01	1.1	7e-02	0.9	8e-02	0.9	4e-03	6
Rru_A1585	Polyhydroxyalkanoate depolymerase PhaZ1	1.9	2e-05	5	0.9	7e-02	1.0	8e-01	1.1	3e-02	1.3	2e-05	6
Rru_A3356	Polyhydroxyalkanoate depolymerase PhaZ2	4.4	7e-03	1	1.0	1e+ 00	1.1	3e-01	0.9	7e-02	0.4	9e-05	1
Rru_A1057	3-hydroxybutyrate dehydrogenase	2.7	1e-06	6	1.0	9e-01	0.9	1e-01	1.3	5e-03	1.1	6e-02	6
Rru_A3695	Acetoacetyl-CoA synthase	2.2	8e-04	6	1.1	7e-02	1.1	2e-02	1.0	7e-01	1.4	8e-05	6
Rru_A3063	Crotonyl-CoA carboxylase/reductase	3.0	4e-04	6	0.7	2e-02	1.0	8e-01	1.0	3e-01	1.8	9e-05	6
Rru_A1572	Ethylmalonyl-CoA/Methylmalonyl-CoA epimerase	0.9	6e-01	5	1.4	2e-02	0.5	1e-04	2.3	6e-07	0.7	3e-03	4
Rru_A3062	Methylmalonyl-CoA mutase (EC 5.4.99.2)	3.7	2e-03	3	1.0	8e-01	1.3	5e-01	1.4	3e-01	1.0	8e-01	2
Rru_A3064	Methylsuccinyl-CoA dehydrogenase	1.5	2e-03	6	0.8	1e-02	0.9	3e-01	1.1	1e+ 00	1.6	3e-05	6
Rru_A1201	Mesaconyl-CoA hydratase	1.5	2e-05	5	1.0	2e-01	1.0	8e-01	1.0	8e-01	1.2	3e-04	6
Rru_A0217	L-Malyl-CoA/b-methylmalyl-CoA lyase	1.0	8e-01	6	1.0	5e-01	1.0	5e-01	1.1	3e-01	1.3	4e-04	6
Rru_A1200	Malyl-CoA thioesterase	1.3	2e-02	6	0.8	2e-03	1.0	8e-01	1.1	9e-02	1.1	3e-02	6
Rru_A0052	Biotin carboxylase	1.2	7e-02	6	0.8	3e-05	0.9	4e-02	1.0	1e-01	1.1	1e-02	6
Rru_A0053	Carboxyl transferase	1.2	8e-03	6	0.8	8e-05	0.9	2e-02	1.0	1e-01	1.1	9e-03	6
Rru_A2479	Methylmalonyl-CoA mutase	1.3	2e-02	5	1.0	8e-01	1.2	1e-01	1.5	3e-01	0.7	6e-01	6
Rru_A1927	Acetyl-CoA hydrolase	4.1	2e-10	6	1.0	5e-01	1.0	9e-01	1.0	1e+ 00	0.5	3e-08	6
Rru_A1205	Succinate dehydrogenase iron-sulfur subunit	1.1	3e-03	5	1.1	1e-01	0.8	4e-03	1.1	6e-02	1.1	5e-01	6
Rru_A1204	Succinate dehydrogenase flavoprotein subunit	1.2	4e-05	5	1.0	6e-01	1.0	1e-01	1.0	3e-02	1.0	8e-02	6
Rru_A1203	Succinate dehydrogenase subunit D	2.0	2e-01	6	1.0	6e-01	1.1	4e-01	0.6	2e-01	1.1	7e-01	1
Rru_A2129	Fumarate hydratase class II	1.9	7e-05	2	1.3	1e+ 00	0.7	1e-01	1.0	1e-01	1.3	2e-01	6
Rru_A2206	Fumarate hydratase class I	1.2	9e-02	6	1.0	4e-01	1.0	1e+ 00	0.9	3e-02	1.0	2e-01	6
Rru_A2398	Pyruvate-flavodoxin oxidoreductase	1.4	4e-03	6	0.9	4e-01	1.0	8e-01	1.3	2e-06	0.8	1e-01	6
Rru_A0695	2-isopropylmalate synthase	1.2	8e-02	6	1.0	3e-01	1.3	3e-02	1.0	6e-01	0.8	4e-01	4
Rru_A1189	3-isopropylmalate dehydratase large subunit	0.8	8e-04	6	1.1	1e-03	1.0	4e-01	1.0	2e-01	0.8	9e-04	6
Rru_A1190	3-isopropylmalate dehydratase small subunit	0.7	1e-02	6	1.2	4e-02	0.9	2e-03	1.1	6e-02	0.9	1e-02	4
Rru_A1191	3-isopropylmalate dehydrogenase	0.9	5e-01	6	1.1	2e-03	0.9	2e-02	1.0	3e-01	0.9	2e-03	5
Rru_A0467	Acetolactate synthase, large subunit	1.5	7e-06	6	1.1	7e-02	0.9	7e-03	0.9	3e-01	1.1	5e-01	6
Rru_A0468	Acetolactate synthase, small subunit	2.0	1e-08	6	1.0	8e-01	1.0	6e-01	0.8	5e-02	1.1	4e-02	6
Rru_A0469	Ketol-acid reductoisomerase (NADP(+))	1.8	1e-05	6	1.0	9e-03	0.9	1e-01	0.9	5e-03	0.9	2e-02	6
Rru_A1786	Dihydroxy-acid dehydratase	1.0	5e-01	6	0.9	1e-02	0.8	7e-02	1.1	5e-01	1.0	6e-01	6
Rru_A2223	Branched chain amino acid aminotransferase	4.3	1e-11	5	0.9	2e-04	0.9	7e-04	0.9	9e-03	0.8	2e-04	6
Rru_A0508	Aminotransferase, class IV	0.3	4e-05	1	2.9	2e-01	0.9	9e-03	1.5	2e-04	1.1	1e-01	1
Rru_A1040	Leucine dehydrogenase	0.1	9e-05	2	0.9	5e-01	0.7	2e-01	2.0	2e-01	2.3	3e-03	2
Rru_A1977	Pyruvate/ketoisovalerate oxidoreductase subunit beta	1.8	1e-02	4	2.3	6e-02	0.8	6e-01	1.2	4e-01	0.3	6e-04	2
Rru_A1978	Pyruvate/ketoisovalerate oxidoreductase subunit alpha	1.9	3e-04	6	1.5	9e-04	0.9	4e-01	1.1	7e-02	0.3	1e-05	6
Rru_A1835	2-methylbutanoyl-CoA dehydrogenase	4.0	6e-06	5	0.8	6e-03	0.8	1e-03	1.3	1e-02	0.9	1e-01	5
Rru_A1833	3-hydroxybutyrate dehydrogenase	2.3	2e-03	5	0.8	2e-03	0.7	7e-04	1.9	3e-06	0.9	1e-02	6
Rru_A1948	Isovaleryl-CoA dehydrogenase	2.8	1e-02	4	0.9	9e-02	0.8	3e-03	1.2	9e-03	0.8	9e-04	4
Rru_A1834	3-hydroxyisobutyryl-CoA hydrolase	2.0	2e-05	5	0.8	2e-02	1.0	9e-01	1.1	2e-01	0.8	9e-04	6
Rru_A1945	Short-chain dehydrogenase/reductase SDR	2.0	3e-07	6	0.8	1e-05	1.0	8e-01	1.0	3e-01	1.2	8e-05	6
Rru_A1946	3-ketoacyl-CoA thiolase	2.3	8e-02	2	0.8	3e-02	0.8	1e-02	1.2	7e-03	0.9	2e-01	3

^a^The protein fold change (FC) is defined as the ratio of the abundance of a protein under different conditions. “B/S” stands for “butyrate vs succinate”. “T1/T0”, “T2/T1”, “T3/T2”

and “T4/T3”’ stand for ratios observed between each culture points of the kinetic proteomic experiment

^b^The statistical significance (*P*-value) of the fold change observed between each condition was determined with an unpaired *t*-test

^c^“pept” represents the number of peptides identified with a confidence higher than 95% and used for quantification

dDue to interference in MS signal, fold change and *P*-value were manually corrected

### Transmission electron microscopy observation of butyrate grown cells

**Fig. 3:**
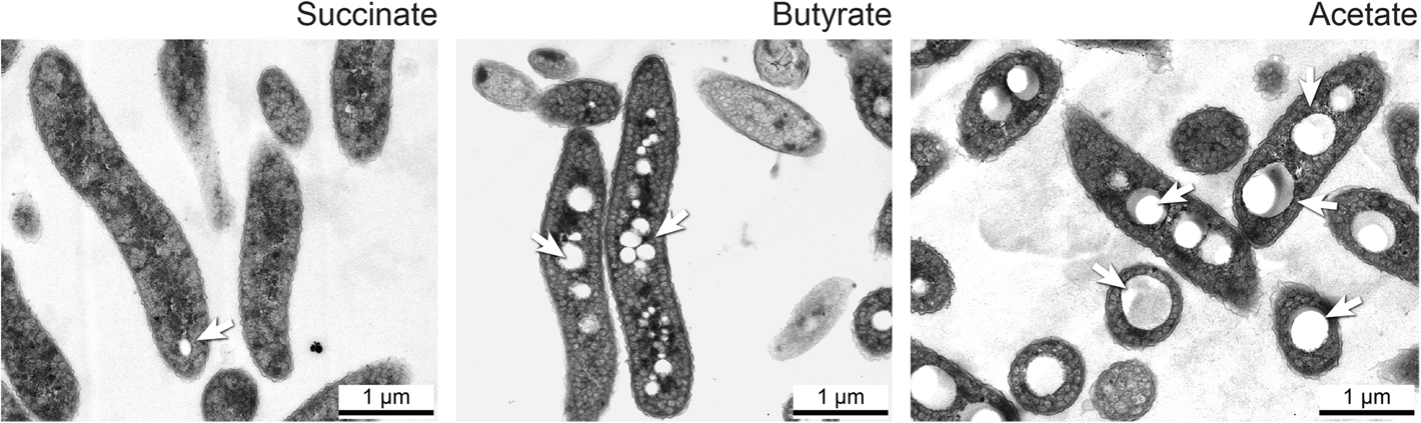
Native PHA (nPHA) granules in *Rs. rubrum S1H* under succinate, butyrate and acetate growth conditions. Transmission electron microphotography of *Rs. rubrum* grown on succinate, acetate and butyrate. Arrows indicate nPHA granules

Our proteomic data revealed that PHA synthesis was potentially upregulated in butyrate condition. In order to validate this hypothesis, we performed transmission electron microscopy on succinate or butyrate grown cultures. Photomicrographs showed higher amount of PHA granules in cells cultivated on butyrate compared to those on succinate (Fig. 3). Compared to cells cultivated under acetate growth conditions characterized by a high production of PHA [10], the granules that appeared under butyrate growth condition were nevertheless smaller and less abundant (Fig. 3).

### Monitoring of the relative abundance of ILV during early growth phase in butyrate condition

**Fig. 4:**
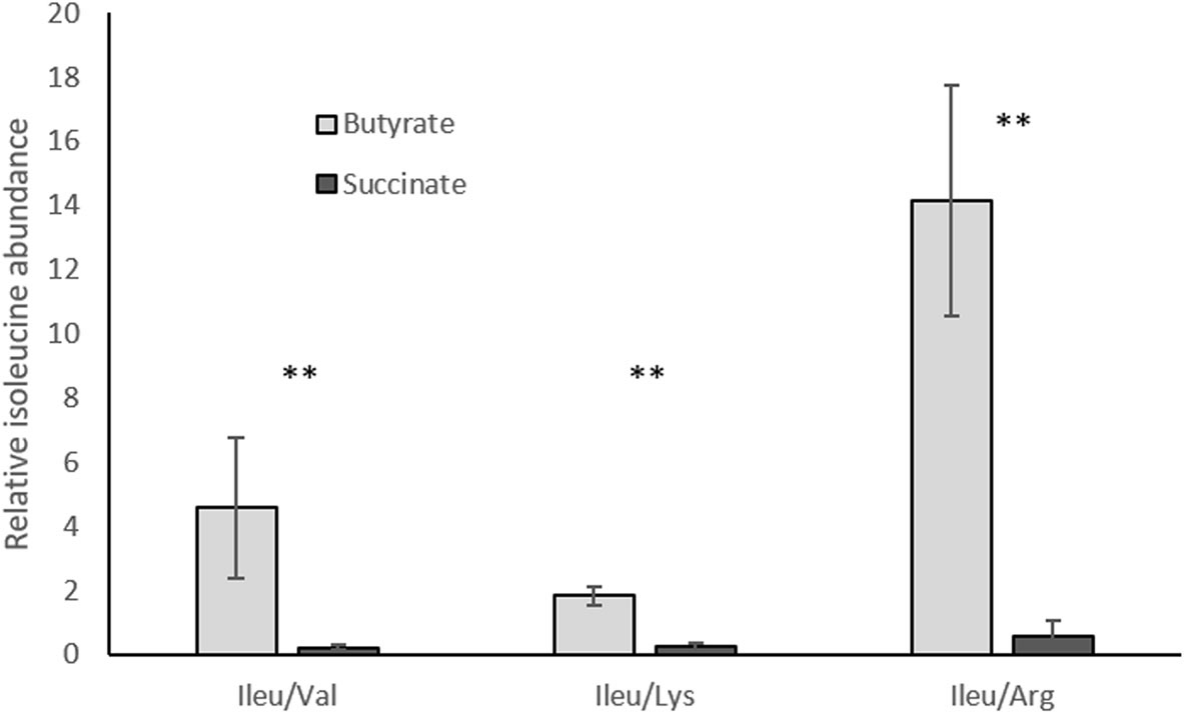
Comparison of the relative abundance of Isoleucine under butyrate and succinate growth condition. Relative abundance of Isoleucine in *Rs. rubrum* was monitored using MRM based mass spectrometry analysis in butyrate (grey) and succinate (black) grown biomass. Isoleucine abundance was expressed as the ratio of isoleucine signal on signal of each of the three other amino acids (valine, lysine and arginine) in order to avoid any extraction bias. (*n* = 5; **: *p* value< 0.01; error bars represent standard error of the mean)

As proteomic data suggested that ILV biosynthesis pathway could be involved in the photoassimilation of butyrate, we determined whether isoleucine or valine were more abundant during the growth in butyrate condition. The relative abundance of these amino acids was measured in the biomass of *Rs. rubrum* growing in succinate or butyrate conditions using targeted mass spectrometry analysis (MRM). As a role in redox balancing is more likely to be observed at the beginning of the growth curve [10, 11], we analyzed samples harvested during the early exponential growth phase (OD_680nm_ ± 0.7). If no difference was observed between succinate and butyrate condition regarding valine content, we observed a large increase in isoleucine relative abundance under butyrate condition (Fig. 4). This relative abundance was calculated as the ratio of the signal obtained for isoleucine over the signal of other amino acids, i.e. valine, lysine or arginine. In all three cases, the difference in abundance of isoleucine was found to be significantly higher under butyrate condition.

### Photoheterotrophic assimilation of ILV by *Rs. rubrum*

Proteomic analysis also revealed that enzymes of the ILV degradation pathway were more abundant under butyrate condition, suggesting this pathway could also be involved in the photoassimilation of butyrate. This pathway was however poorly annotated in *Rs. rubrum* and we thus decided to test the capacity of *Rs. rubrum* to effectively degrade ILV under photoheterotrophic conditions. We tested the growth of our strain in a medium composed of succinate or acetate supplemented with 30 mM ILV mixture (10 mM each). In medium supplemented with succinate or acetate only, the culture reached an optical density of 4.5, which corresponded to the exhaustion of the carbon source (Fig. S3). On the other hand, the addition of ILV allowed growth to continue until an optical density close to 10 was reached, suggesting that ILV can serve as a carbon source for photoheterotrophic growth of *Rs. rubrum* (Fig. S3). This result confirms that our strain is able to assimilate ILV as carbon source under photoheterotrophic conditions.

### Kinetic proteomic analysis of butyrate assimilation

**Fig. 5:**
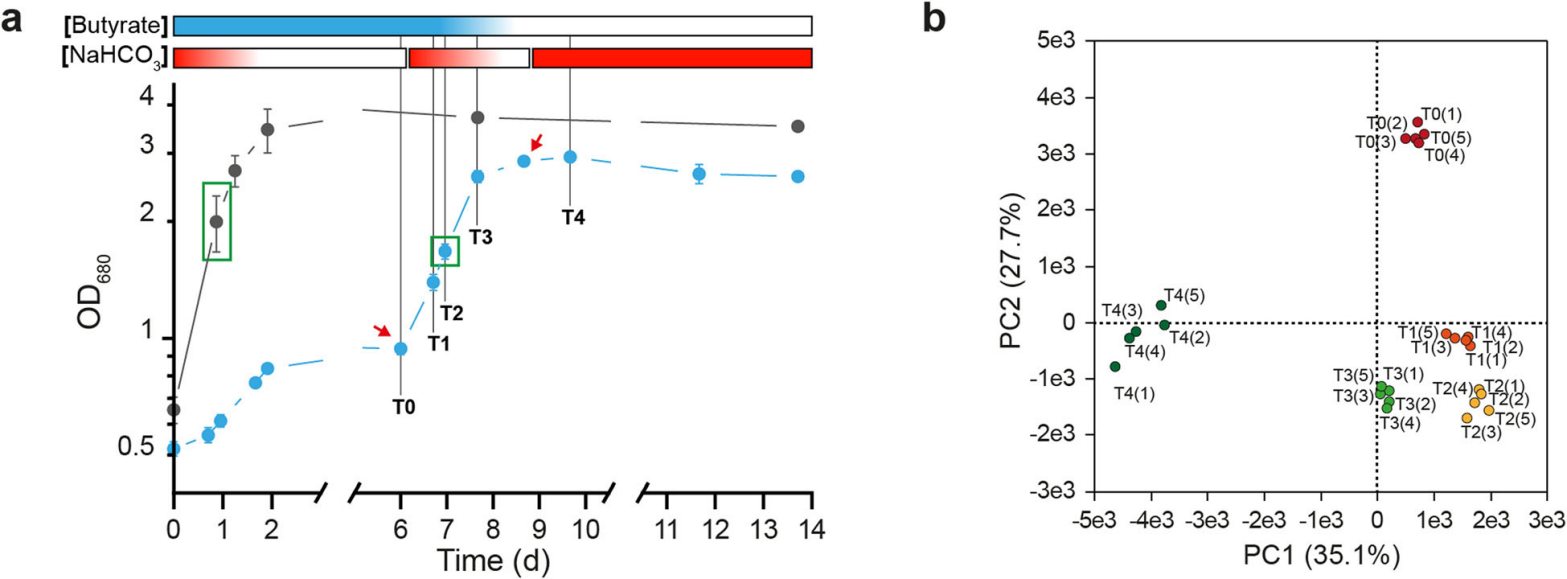
Kinetic proteomic analysis of *Rs. rubrum* S1H cultivated under butyrate growth conditions. **a** Representation of the different sampling points used for kinetic proteomic analysis (T0, T1, T2, T3, and T4; *n* = 5). Growth in the presence of succinate (gray; *n* = 5) or butyrate (blue; n = 5) was monitored by measuring OD_680nm_. Red arrows represent bicarbonate supplementation (180 μmoles of NaHCO_3_, 6.5 ± 0.5 mM final). The color gradients above represent the expected concentration of butyrate (blue) and carbonate (red) in the culture medium, from most concentrated (color) to less concentrated (white). Green boxes indicate samples used for the first proteomic experiment comparing growth in medium supplemented with butyrate or succinate. **b** Principal component analysis of the proteomic data obtained from the kinetic proteomic experiment. Red, orange, yellow, light green and dark green dots represent the culture samples from the T0, T1, T2, T3 and T4 time points, respectively. Each axis represents a principal component (PC1 and PC2) with the percentage of the total variance it explains. The next two components (PC3 and PC4) explained 9.0 and 6.5% of the total variance, respectively

As EMC and ILV synthesis/degradation pathways were highlighted by our proteomic analysis as being potentially involved in butyrate photoassimilation, we decided to test if these pathways could be used sequentially, in response to different environmental conditions occurring during the growth curve. To test this hypothesis, we measured the relative abundance of proteins at different phases of growth along a growth curve under butyrate condition. The analyzed time points were selected to represent different availability of nutrients and are described in Fig. 5a. After the first growth arrest in medium supplemented with butyrate and a limiting amount of bicarbonate (T0), we added bicarbonate to stimulate growth restart (T1-T2-T3) and followed the growth until the stationary phase (T4). These 5 time points cover 3 different growth conditions: (1) butyrate available and bicarbonate unavailable (T0), (2) butyrate and bicarbonate available (decreasing concentrations) (T1, T2, T3, 3) butyrate unavailable and bicarbonate available (T4). Excess bicarbonate was added to the culture after the first characteristic growth arrest to obtain continuous growth covering these different conditions. This kinetic proteomic analysis resulted in the identification (at a global false discovery rate below 1%) and quantification of 1804 proteins. The complete data set is available in the supporting material (Table S3), and the discussed data are listed in Table 1. A principal component analysis (PCA) was also performed on the proteomic data of the 5 culture points described above (Fig. 5b). The first principal component (PC1; 35.1% of the total variance) clearly isolated the last culture point (T4) while the second principal component (PC2; 27.7% of the total variance) isolated the first culture point (T0), clearly separating the data in 3 groups corresponding to the 3 different growth conditions previously described. The results obtained in this kinetic proteomic analysis are further discussed in the following section.

### Genome wide mutant fitness assay under butyrate condition

**Fig. 6:**
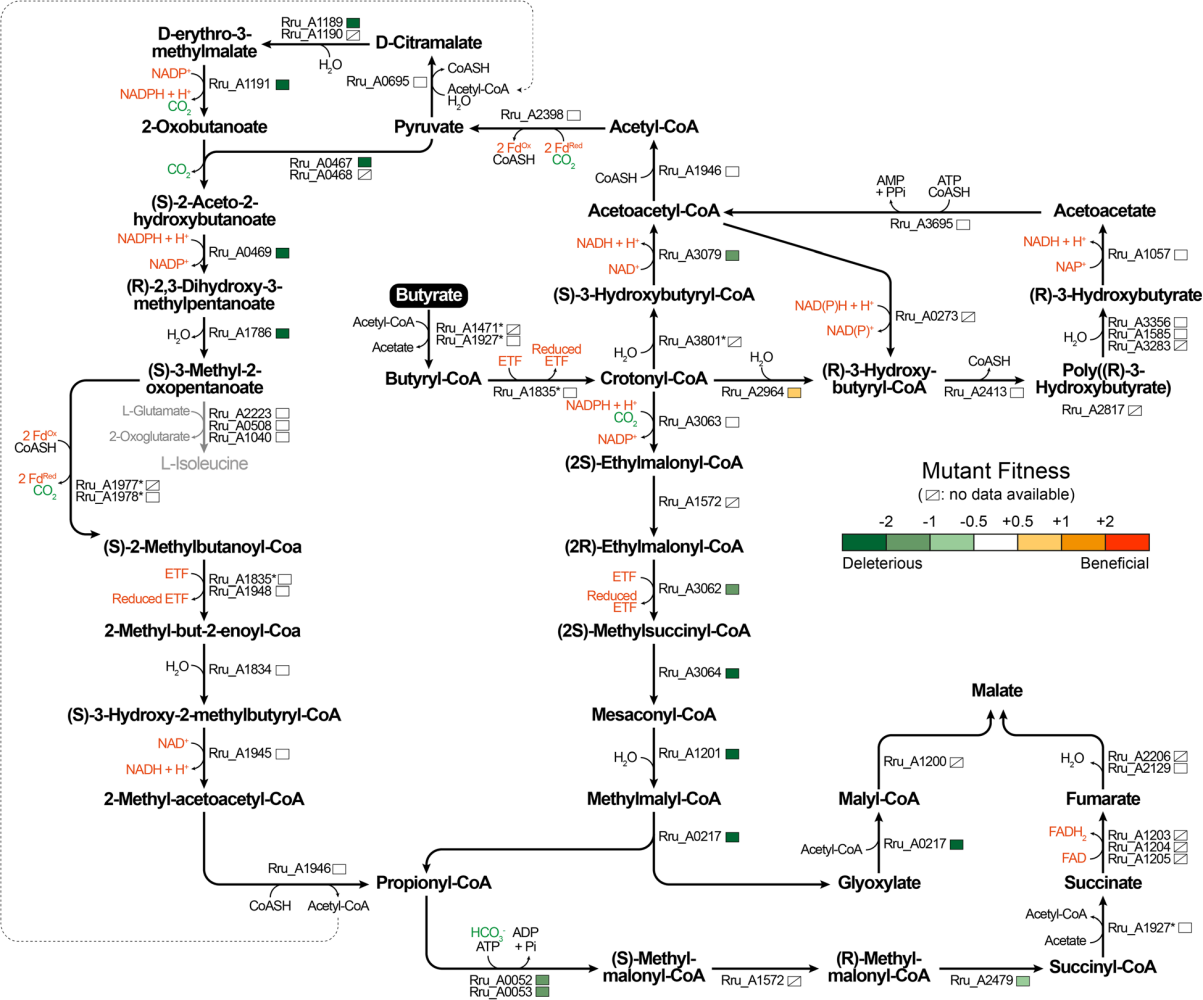
Fitness profiling of a *Rs. rubrum* transposon mutant library grown on butyrate. The colored markers indicate the fitness values of strains with mutation in genes encoding related enzymes, ranging from green (deleterious mutation; gene required for optimal growth on butyrate) to orange (beneficial mutation; gene deleterious for optimal growth on butyrate). Strikethrough markers stand for unavailable data due to exclusion according to our robustness criteria or due to the absence of mutated strains in the mutant library. The enzymes marked with a star have yet to be confirmed due to either possible multiple assignations in proposed pathways or to uncertain enzymatic activity. ETF stands for electron transfer flavoproteins. Fd stands for ferredoxins

To further explore the requirement of the EMC and MBC pathways for optimal growth under butyrate condition, we used genome-wide mutant fitness assays to probe the importance of most *Rs. rubrum* genes in this condition. Here, we leveraged a *Rs. rubrum* randomly barcoded transposon mutant library (RB-TnSeq) that permits the parallel assessment of gene fitness using a quantitative DNA barcode sequencing strategy [12] and has already been successfully used to study the assimilation of acetate [13]. Of the 2138 genes for which fitness data were obtained, only 37 genes presented a fitness effect that was specifically associated with the adaptation to photoheterotrophic assimilation of butyrate. The complete data set is available in Supporting Information (SI) Table S4, and the discussed results are presented in Table 2. Figure 6 illustrates the main finding of this experiment. Interestingly, if most genes of the EMC pathway appeared to be essential for optimal growth under butyrate conditions, *ccr*, a key gene of this pathway, was not essential. The detailed interpretation of the results is given in the discussion section.

**Table 2:** Gene fitness values of selected genes in *Rhodospirillum rubrum S1H*

Locus Tag	Description	Succinate gene fitness^**c**^	Butyrate gene fitness^**c**^	***P***-value^**2**^
Rru_A1471	Butyryl-CoA:acetate CoA transferase	n.a.	n.a.	n.a.
Rru_A1927	Acetyl-CoA hydrolase	− 0.01	0.1	3e-01
Rru_A3801^b^	Short chain enoyl-CoA hydratase	n.a.	n.a.	n.a.
Rru_A3079	3-hydroxyacyl-CoA dehydrogenase	−0.45	− 1.16	2e-02
Rru_A1946	Acetoacetyl-CoA thiolase	0.06	−0.03	7e-01
Rru_A0273^b^	Acetoacetyl-CoA reductase	n.a.	n.a.	n.a.
Rru_A2964	(R)-specific enoyl-CoA hydratase	−0.04	0.79	9e-05
Rru_A2413	Poly(R)-hydroxyalkanoic acid synthase	−0.48	0.11	3e-02
Rru_A2817^b^	Phasin	n.a.	n.a.	n.a.
Rru_A3283^a^	Activator of polymer mobilization	n.a.	n.a.	n.a.
Rru_A1585	Polyhydroxyalkanoate depolymerase PhaZ1	−0.04	− 0.05	7e-01
Rru_A3356	Polyhydroxyalkanoate depolymerase PhaZ2	0.03	−0.42	5e-03
Rru_A1057	3-hydroxybutyrate dehydrogenase	−0.11	−0.48	9e-02
Rru_A3695	Acetoacetyl-CoA synthase	−0.17	−0.41	2e-02
Rru_A3063	Crotonyl-CoA carboxylase/reductase	−0.15	−0.18	7e-01
Rru_A1572^a^	Ethylmalonyl-CoA/Methylmalonyl-CoA epimerase	n.a.	n.a.	n.a.
Rru_A3062	Methylmalonyl-CoA mutase (EC 5.4.99.2)	0.02	−1.77	5e-04
Rru_A3064	Methylsuccinyl-CoA dehydrogenase	−0.01	−2.3	1e-03
Rru_A1201	Mesaconyl-CoA hydratase	0.06	−3.42	2e-07
Rru_A0217	L-Malyl-CoA/b-methylmalyl-CoA lyase	−0.05	−3.11	1e-05
Rru_A1200^b^	Malyl-CoA thioesterase	n.a.	n.a.	n.a.
Rru_A0052	Biotin carboxylase	−0.03	−1.83	2e-05
Rru_A0053	Carboxyl transferase	−0.09	−1.77	2e-04
Rru_A2479	Methylmalonyl-CoA mutase	−0.12	−0.53	1e-02
Rru_A1927	Acetyl-CoA hydrolase	−0.01	0.1	3e-01
Rru_A2129	Fumarate hydratase class II	0.15	−0.03	1e-01
Rru_A2206	Fumarate hydratase class I	n.a.	n.a.	n.a.
Rru_A2398	Pyruvate-flavodoxin oxidoreductase	−0.15	−0.17	6e-01
Rru_A0695	2-isopropylmalate synthase	−0.08	−0.06	8e-01
Rru_A1189	3-isopropylmalate dehydratase large subunit	−3.87	−3.24	1e-02
Rru_A1190	3-isopropylmalate dehydratase small subunit	n.a.	n.a.	n.a.
Rru_A1191	3-isopropylmalate dehydrogenase	−3.32	− 3.17	8e-01
Rru_A0467	Acetolactate synthase, large subunit	−1.95	−3.46	1e-02
Rru_A0468^b^	Acetolactate synthase, small subunit	n.a.	n.a.	n.a.
Rru_A0469	Ketol-acid reductoisomerase (NADP(+))	−2.21	−3.31	3e-02
Rru_A1786	Dihydroxy-acid dehydratase	−2.35	−3.49	5e-02
Rru_A2223	Branched chain amino acid aminotransferase	−0.11	0.01	3e-01
Rru_A0508	Aminotransferase, class IV	0.02	0.1	5e-01
Rru_A1040	Leucine dehydrogenase	0.05	0.03	8e-01
Rru_A1977	Pyruvate/ketoisovalerate oxidoreductase subunit beta	n.a.	n.a.	n.a.
Rru_A1978	Pyruvate/ketoisovalerate oxidoreductase subunit alpha	−0.01	−0.07	5e-01
Rru_A1835	2-methylbutanoyl-CoA dehydrogenase	−0.02	0.08	6e-01
Rru_A1948	Isovaleryl-CoA dehydrogenase	−0.01	0	9e-01
Rru_A1834	3-hydroxyisobutyryl-CoA hydrolase	−0.15	0.04	5e-01
Rru_A1945	Short-chain dehydrogenase/reductase SDR	0.09	−0.04	2e-01
Rru_A1946	3-ketoacyl-CoA thiolase	0.06	−0.03	7e-01

^a^Central sequence too short to be included in data set based on acceptance criteria used in this study

^b^Not included in the dataset because fitness could only be accurately measured in 2 out of 3 of the replicates

^c^The strain fitness is defined as the log_2_ of the ratio between the strain abundance reached after 5 generations to the abundance at T_0_ in the relevant condition. The gene fitness values are calculated by averaging the strain fitness values for each gene. The presented gene fitness values are the average values resulting of three independent fitness assays for each condition. The statistical significance (*P*-value) between the acetate and succinate condition was determined with an unpaired *t*-test. The complete data set is available in supplemental material

### Role of Crotonyl-CoA carboxylase/reductase (ccr) in butyrate photoassimilation

**Fig. 7:**
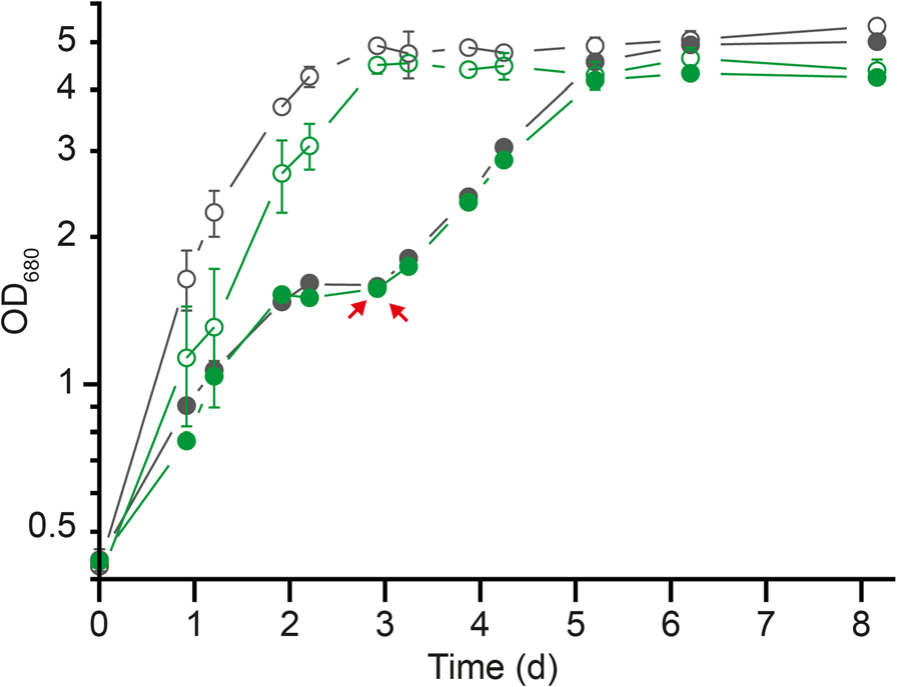
Targeted mutagenesis against the crotonyl-CoA carboxylase/reductase gene (*ccr*). The growth of the *Rs. rubrum* S1H ∆*ccr*::Km^R^ (green; n = 5) and wild-type (gray; n = 5) strains was monitored by measuring the OD_680nm_ under light anaerobic conditions with succinate (opened markers) or butyrate (filled markers) as the carbon source and supplemented with 50 mM bicarbonate. Red arrows represent bicarbonate supplementation (NaHCO_3,_ 180 μmoles)

To validate the results of the genome wide mutant fitness assay, we tested the growth capacity of our (∆*ccr*::Km^R^) mutant under butyrate condition. This mutant was recently shown to be unable to grow under photoheterotrophic conditions with acetate as the sole carbon source [13]. The phenotypic characterization of this mutant under photoheterotrophic conditions confirmed the results of the genome wide mutant fitness assay with RB-TnSeq, as there was no significant phenotypic variation between *wt* and mutant strain while growing in medium supplemented with succinate or butyrate (Fig. 7). As *rru_A3063* is clustered with *rru_A3062* and *rru_A3064* (Fig. S1), the *ccr* mutation could also impair the expression of *rru_A3062* and *rru_A3064*. To test this hypothesis, a targeted proteomic analysis using the MRM method was performed to quantify the abundance of the Rru_A3062, Rru_A3063, Rru_A3064 proteins in wild-type and ∆*ccr*::Km^R^ strains growing on butyrate (Supplementary results). The results of this targeted analysis revealed that our mutation had no effect on the abundance of Rru_A3062 (Fig. S2). However, if Rru_A3064 was still expressed in the *ccr* mutant strain, its abundance decreased by 60%.

## Discussion

The photoheterotrophic growth of *Rhodospirillaceae* on highly reduced substrates such as butyrate or propionate has been known to require bicarbonate in the culture medium [14]. In *Rhodobacter capsulatus*, another PNSB, growth on butyrate without CO_2_ supplementation is only observed in conditions permitting H_2_ production [15] or if auxiliary electron acceptor as NO_3_, N_2_O, DMSO or TMAO [16] are present. Consequently, it was suggested that CO_2_ fixation could act as an electron sink allowing the recycling of reduced cofactors and maintaining an appropriate redox balance to support the photoassimilation of highly reduced substrates [17]. The dependency of butyrate assimilation on bicarbonate observed here most likely reflects the redox imbalance triggered by this highly reduced carbon source.

Proteomic analysis only allows the evaluation of changes in the abundance of proteins which does not always imply a change in the activity or flux of a pathway. Nevertheless, based on our proteomic analysis, we propose the potential assimilation pathways depicted in Fig. 2 and discussed hereafter. These findings will require further studies, notably through metabolic flux analysis, to determine the actual use of these pathways.

### Butyrate is partly converted into acetyl-CoA

The first step involved in the assimilation of butyrate is its activation in butyryl-CoA (Fig. 2). One of the most likely activating enzymes is the butyryl-CoA:acetate CoA transferase (Rru_A1471). A homologue of this enzyme has already been proposed as the enzyme that activates butyrate during its catabolism in *Desulfosarcina cetonica* [4]. However, this enzyme was quantified with only one peptide in our dataset and showed a slightly but not significantly higher abundance under butyrate growth conditions (fold change, 1.5; *P*-value, 6.7e-1). Alternatively, Rru_A1927, annotated as an acetyl-CoA hydrolase, was strongly upregulated (fold change, 4.1; *P*-value, 2.3e-10) under butyrate growth condition and could also be involved in butyrate activation. According to the InterPro database [18], Rru_A1927 is related to the succinyl-CoA:coenzymeA transferase (2.3.8.-) family. Depending on its specificity, it could therefore directly transfer CoA from succinyl-CoA to butyrate and as such be the butyrate-activating enzyme.

The butyryl-CoA is then oxidized to crotonyl-CoA through a butyryl-CoA dehydrogenase activity. Rru_A1835 is highly upregulated in the presence of butyrate (fold change, 4.0; *P*-value, 5.6e-6) and is annotated as a butyryl-CoA dehydrogenase. Rru_A1835 is also the best homologue, in the *Rs. rubrum* genome, of the butyryl-CoA dehydrogenase reviewed in *Clostridium acetobutylicum* [19] (accession number P52042) or *Megasphaera elsdenii* [20] (accession number Q06319).

As already described in *Desulfosarcina cetonica* [4] in the context of butyrate catabolism, crotonyl-CoA is proposed to be further transformed into acetyl-CoA (Fig. 2). It is first mainly converted to (S)-3-hydroxybutyryl-CoA by a 3-enoyl-CoA hydratase, most likely by Rru_A3801, which was only slightly upregulated (fold change, 1.2; *P*-value, 9.8e-3) under butyrate growth conditions. A 3-hydroxybutyryl-CoA dehydrogenase (Rru_A3079; fold change, 1.9; *P*-value, 5.3e-8) catalyzes the oxidation of (S)-3-hydroxybutyryl-CoA to acetoacetyl-CoA which finally undergoes a thioclastic cleavage, releasing two molecules of acetyl-CoA. The acetoacetyl-CoA thiolase (Rru_A1946; fold change, 3.7; *P*-value, 2.2e-2) is probably the best candidate to catalyze this last step. Other acetyl-CoA C-acetyltransferases (Rru_A0274, Rru_A3387 and Rru_A1310) were identified in our dataset but were downregulated or not significantly upregulated under butyrate growth condition.

### PHA are produced during butyrate photoassimilation

The conversion of crotonyl-CoA to (R)-3-hydroxybutyryl-CoA is known to be the entry point for the poly (3-hydroxybutyrate) (PHB) biosynthesis pathway [21, 22]. Even if PhaJ (Rru_A2964) abundance was not specifically affected when butyrate is the carbon source (fold change, 0.9; *P*-value, 4.8e-1), this protein is probably involved in the maintenance of this route. Conversely, an (R)-specific acetoacetyl-CoA reductase (Rru_A0273) was strongly downregulated under butyrate growth conditions (fold change, 0.3; *P*-value, 4.2e-7). Beyond its (R)-isomeric specificity, this enzyme was also associated with acetate photoassimilation, catalyzing the reaction in the reverse direction [10]. The specific up- and downregulation of this enzyme under acetate and butyrate growth conditions, respectively, and the reverse situation observed for Rru_A3079 probably highlight a substrate preference for each enzyme, one favoring the conversion of acetoacetyl-CoA to 3-hydroxybutyryl-CoA during acetate assimilation, the other favoring the reverse reaction when butyrate is used as unique carbon source.

The production of (R)-3-hydroxybutyryl-CoA was therefore maintained under butyrate growth condition and sustained PHB biosynthesis (Fig. 2). The poly(R)-3hydroxyalkanoic acid synthase PhaC2 (Rru_A2413) was slightly more abundant under butyrate growth condition (fold change, 1.7; *P*-value, 1.1e-4). Phasin (Rru_A2817), constituting the protein layer on the surface of PHB granules was also strongly upregulated (fold change, 49.8; *P*-value, 5.0e-6). Higher production of PHA granules under butyrate conditions was beside confirmed by transmission electron microscopy.

Surprisingly, enzymes responsible for the mobilization of PHB were also significantly upregulated (Fig. 2). The intracellular PHB depolymerase PhaZ2 (Rru_A3356) was clearly upregulated (fold change, 4.4; *P*-value, 6.9e-3). PhaZ1 (Rru_A1585; fold change, 1.9; *P*-value, 1.7e-5) and its phasin-like activator [23, 24] ApdA (Rru_A3283; fold change, 9.7; *P*-value, 6.0e-6) were also more abundant under butyrate growth condition. PhaZ1 was described as being highly specific to native PHB granules and short chain-length PHAs while being periplasmic and connected with the extracellular medium [25]. Its cellular function remains enigmatic and its involvement in butyrate metabolism is therefore not clear. Finally, enzymes responsible for the conversion of 3-hydroxybutyrate into acetoacetyl-CoA (Rru_A1057 and Rru_A3695) were quantified with a higher abundance under butyrate growth condition. The simultaneous PHB synthesis and degradation as well as the constitutive expression of PHB synthase and depolymerase were already reported in *Ralstonia eutropha* [26–28]. This phenomenon, for which physiological signification has yet to be found, thus also potentially occurs in *Rs. rubrum* when butyrate is used as the sole carbon source. In the context of acetate assimilation, the biosynthesis of PHB from acetyl-CoA has been proposed to have a redox balancing role through the reduced equivalent-consuming conversion of acetoacetyl-CoA to 3-hydroxybutyryl-CoA [10, 29]. In the context of butyrate assimilation, and the production of PHB from crotonyl-CoA, the electron sink function of the PHB biosynthesis pathway is not applicable (Fig. 2) and the direct mobilization of PHB leads to the same metabolic intermediate (i.e., acetoacetyl-CoA) with a higher energy cost. The upregulation of this apparently futile cycle during butyrate assimilation remains then largely misunderstood. One can speculate that the use of PHB biosynthesis here could serve as a “buffer zone” to limit the carbon flow and regulate the reduced equivalent production.

### The ethylmalonyl-CoA pathway is active under butyrate growth conditions

Under photoheterotrophic conditions, the conversion of butyrate into acetyl-CoA necessarily implies the reassimilation of the latter through an anaplerotic pathway to maintain the pool of TCA cycle intermediates required as biosynthetic precursors. Interestingly, the first intermediate of butyrate photoassimilation, crotonyl-CoA, is also the first intermediate of the ethylmalonyl-CoA pathway, a C2 compound anaplerotic pathway [6–8, 10]. Based on our proteomic data, the EMC pathway could also be used under butyrate growth conditions. Indeed, except for the ethylmalonyl/methylmalonyl-CoA epimerase (Rru_1572), all proteins of the EMC pathway (Rru_A3063, Rru_A3062, Rru_A3064 and Rru_A1201) were observed in higher abundance under butyrate conditions (Fig. 2) (Table 1).

In the second part of the EMC pathway, the enzymes of the methylmalonyl-CoA pathway are responsible for the conversion of propionyl-CoA to succinyl-CoA. These enzymes (Rru_A0052, Rru_A0053, Rru_A1572 and Rru_A2479) were also expected to be upregulated, as recently observed with acetate as a carbon source [10]. However, only some of those enzymes appear to be slightly upregulated under butyrate growth conditions compared to succinate. The apparent lack of upregulation observed here remains misunderstood since the methylmalonyl-CoA pathway is expected to directly connect the EMC pathway to central carbon metabolism. Globally, the result of our proteomic analysis may indicate a limited involvement of the EMC pathway in butyrate assimilation.

### The branched chain amino acids biosynthesis pathway is upregulated under butyrate conditions

In our previous study, we highlighted the probable involvement of the branched chain amino acids (isoleucine, leucine and valine, ILV) biosynthesis pathway in acetate photoassimilation [10]. The acetate-specific upregulation of the enzymes catalyzing this pathway, except for the final transaminases (the abundances of which were significantly decreased), suggested that this route has another outcome than ILV biosynthesis. Under butyrate growth conditions, we again observed a clear upregulation of this pathway, starting from Rru_A2398 and, more specifically, the L-valine/L-isoleucine biosynthesis pathway. Except for IlvD (Rru_A1786), all the enzymes (Rru_A2398, Rru_A0467, Rru_A0468, Rru_A0469) responsible for the conversion of acetyl-CoA into 2-oxoisovalerate or (S)-3-methyl-2-oxopentanoate were significantly more abundant under butyrate growth conditions (Fig. 2) (Table 1). As already observed in the case of acetate metabolism, leucine dehydrogenase (Rru_A1040), the enzyme catalyzing the final step of branched chain amino acid biosynthesis, was highly downregulated with butyrate as the carbon source. Nevertheless, other aminotransferases (EC 2.6.1.42) were identified in the *Rs. rubrum* genome, some of which were downregulated (Rru_A0508), while others were more abundant under butyrate conditions (Rru_A2223). The poor annotation of these enzymes and the lack of information regarding their specificity make interpretation of these results complicated. Interestingly, a recent study by McCully et al. [30] concluded that reductive amino acid synthesis and, in particular, isoleucine synthesis could be used by *Rs. rubrum* as an electron balancing mechanism during malate and fumarate assimilation. In our case, starting from butyrate, isoleucine synthesis could be considered reductive (Fig. 2) and could thus serve as an electron balancing function. ILV biosynthesis pathway was also recently observed as upregulated in response to light stress under acetate condition in our lab [11] and a role in redox balancing was also suggested.

The results of the monitoring of the relative abundance of isoleucine in the biomass during the early phase of growth under butyrate condition also claim for the involvement of isoleucine production in the assimilation of butyrate, at least during the early growth phase. If this production of isoleucine effectively plays a role in terms of redox balancing will require furthers investigations.

### The branched chain amino acids (ILV) degradation pathway is upregulated

Surprisingly, our proteomic data revealed a concomitant upregulation of some enzymes involved in the ILV degradation pathway. Although the existence of the ILV degradation pathway has been known for a long time [31, 32], this pathway remains unclear or badly annotated in *Rs. rubrum* and requires some clarifications. To detect genes belonging to the ILV degradation pathway in *Rs. rubrum*, we searched in the genome of *Rs. rubrum* for the ILV box, the consensus DNA sequence that the regulator of the ILV degradation genes binds to [33]. The ILV box could be found upstream of *rru_A1994*, annotated as a *merR*-like transcription factor. Using blast, we observed that *rru_A1994* is the best homologous sequence for *liuR* of *Sh. oneidensis* (Table S2). The ILV degradation pathway has been well characterized in this species [33]. Rru_A1994 should thus be annotated as LiuR and is probably the regulator of the ILV degradation pathway in *Rs rubrum*. Rru_A1994 was clearly upregulated when butyrate was used as sole the carbon source.

The ILV box could also be detected upstream of the cluster *rru_A1835*, *rru_A1834* and *rru_A1833* which, based on sequence comparison with *Sh. oneidensis* data, correspond to *ivdC*, *ivdE* and *ivdF* respectively. The *ivd* gene cluster encodes proteins specifically responsible for L-isoleucine and L-valine degradation. Rru_A1835, Rru_A1834 and Rru_A1833 were all clearly upregulated in cultures grown in the presence of butyrate (Table 1). Some elements of the *Sh. oneidensis ivd* cluster, *ivdB, ivdD* and *ivdG,* are missing in the *ivd* cluster of *Rs. rubrum*. To identify these missing elements, we blasted the sequence of *Sh. oneidensis* genes against the *Rs. rubrum* genome. The best homologues for those genes in the *Rs.rubrum* genome were identified (*rru_A3801*, *rru_A1507*, *rru_A2071*/*rru_A1542*; Table S2) but were not differentially regulated under butyrate condition or were even in lower abundance. Importantly, the ILV box could not be detected in the neighborhood of those external genes.

The ILV box was finally also detected upstream of the cluster of genes from *rru_A1940* to *rru_A1948*. This cluster of genes corresponds to the *liu* cluster of *Sh. oneidensis,* which contains genes encoding proteins involved in L-leucine degradation. Among the homologues of Liu proteins in this cluster, only Rru_A1948 and Rru_A1942, which correspond to LiuA and LiuC of *Sh. oneidensis*, respectively, were detected with a higher abundance in the butyrate-assimilating cultures. Rru_A1943, Rru_A1941 and Rru_A1940, corresponding to LiuB, LiuD and LiuE, respectively, could not be detected at the protein level. The homologues of LiuF and LiuG could not be found in this cluster and were identified as Rru_A1383 and Rru_A1382 by blast. These two proteins could also not be identified in our proteomic data, and no ILV box could be detected in the neighborhood of their coding genes. On the other hand, four other proteins of the cluster Rru_A1940-Rru_A1948 were detected in higher abundance under butyrate conditions. Rru_A1947 and Rru_A1944 are annotated as nucleotidyltransferase-like and DSBA oxidoreductase, respectively, and no function in ILV degradation could be identified for these proteins. Rru_A1945 is annotated as a short chain dehydrogenase, which is a large family of enzymes that also contains hydroxyl-acyl dehydrogenase. Even though the best homologue for IvdG of *Sh. oneidensis* in the *Rs. rubrum* genome was Rru_A1507, Rru_A1945 presented some similarities with this protein (Identities: 28%, Positives: 48%, Table S2). Rru_A1945 could thus be the 3-hydroxyacyl-CoA dehydrogenase responsible for the reduction of 3-hydroxy-2-methylbutyryl-CoA to 2-methyl-acetoacetyl-CoA, but this annotation would require further investigation.

The L-valine/L-isoleucine degradation pathway usually leads to the production of propionyl-CoA, which is then assimilated through the methylmalonyl-CoA pathway. In the case of L-valine degradation, the last intermediate is the methylmalonate-semialdehyde, which requires dehydrogenase to be converted to propionyl-CoA. Rru_A1542 and Rru_A2071 were identified as the best candidates to catalyze this step in *Rs. rubrum*, as they were the best homologues of IvdB of *Sh. oneidensis*. However, both enzymes seemed to be strongly downregulated under butyrate growth condition. On the other hand, the last intermediate of L-isoleucine degradation is the 2-methylacetoacetyl-CoA, which is converted to propionyl-CoA by a thiolase. This thiolase is coded by the *ivdA* gene in *Sh. oneidensis*. Interestingly, the best homologue candidate for IvdA in *Rs. rubrum* is Rru_A1946 (60% identity) which was observed in higher abundance in the butyrate assimilating cultures. The thiolase coded by *rru_A1946* links L-isoleucine degradation pathway to the methylmalonyl-CoA pathway.

Altogether, these data suggest that the ILV degradation regulon, regulated by LiuR via the ILV box, is involved in butyrate assimilation in *Rs. rubrum*. The ILV box could also be detected downstream of the leucine dehydrogenase-encoding gene Rru_A1040, which was highly downregulated under butyrate conditions, also suggesting the involvement of a global regulatory mechanism. The organization of the *liu* and *ivd* operons seems to be largely different in *Rs. rubrum* than in other proteobacteria. In particular, the genes of the *ivd* cluster are present in the *liu* operon, and some genes, such as *liuFG* and *ivdB* and *ivdD,* are not dependent on the ILV box. It is currently unknown whether this organization is specific to *Rs. rubrum* or generalized to α-proteobacteria.

Based on these results, we could thus reconstruct the complete L-isoleucine degradation pathway (Fig. 2) of *Rs. rubrum*. Our proteomic data show that all the proteins involved in the L-isoleucine degradation pathway were present in higher abundance when butyrate was used as the sole carbon source, suggesting the involvement of the isoleucine biosynthesis and degradation in butyrate assimilation. It is important to mention that the role of ILV synthesis and degradation is not fully understood in purple bacteria. A recent study in *Rh. capsulatus* highlighted that genes of the ILV degradation such as mmsA,B; isobutyryl-CoA dehydrogenase; 3-hydroxyisobutyryl-CoA hydrolase; mccA,B, hmglL or menB were downregulated in a mutant lacking AerR, which acts as an indirect photosynthesis activator. This observation already linked ILV degradation metabolism with photosynthetic metabolism in another purple bacteria [34].

It can also not be ruled out that the production of branched chain amino acids also plays a role in signaling as valine and leucine were reported to be involved in alarmones regulation and in particular in development of the stringent response [35].

### Butyrate cannot directly enter ILV degradation pathway

Our proteomic data suggest that the L-isoleucine biosynthesis and degradation pathways are upregulated when butyrate is used as a unique carbon source. Surprisingly, while the class IV aminotransferase Rru_A0508 and the L-leucine dehydrogenase Rru_A1040, both of which are involved in the final transamination of the ILV biosynthesis pathway, were clearly downregulated under butyrate growth condition, a third enzyme, the branched-chain-amino-acid aminotransferase Rru_A2223, was conversely upregulated (fold change, 4.3; *P*-value, 1.0e-11). This result is paradoxical in the context of a concomitant upregulation of the biosynthesis and the degradation pathways of ILV. A decoupling of these two pathways was considered and a hypothetical isobutyryl-CoA mutase (ICM) able to directly convert butyryl-CoA to isobutyryl-CoA as described in *Streptomyces cinnamonensis* [36, 37] was searched in the *Rs. rubrum* genome. Sequence comparison of the coenzyme B12-dependent isobutyryl-CoA mutase of *S. cinnamonensis* showed 43% identity (E-value: 5e-137) with Rru_A2479, a methylmalonyl-CoA mutase that apparently catalyzes the conversion of (R)-methylmalonyl-CoA to succinyl-CoA in *Rs. rubrum*. Nevertheless, Rru_A2479 showed 79% identity (E-value: 0.0) with the closely-related methylmalonyl-CoA mutase of *S. cinnamonensis*, which was reported to exhibit no isobutyryl-CoA mutase activity [38]. In addition, based on bioinformatics analysis, Jost et al. [39] could highlight two specificity determinant sites in the sequence of acyl-CoA mutase family of protein which both confirmed that Rru_A2479 belongs to the methymalonyl-CoA mutase (Tyr-92 and Arg-210). The presence of an isobutyryl-CoA mutase in the *Rs. rubrum* genome is therefore unlikely and the upregulation of the branched-chain-amino-acid aminotransferase Rru_A2223 remains puzzling.

### ILV biosynthesis and ILV degradation pathways could be directly coupled

On the other hand, 3-methyl-2-oxopentanoate is also the first intermediate in the catabolism of L-isoleucine, a 3-methyl-2-oxopentanoate dehydrogenase could be the connecting enzyme between the L-isoleucine biosynthesis and degradation pathways (Fig. 2), converting 3-methyl-2-oxopentanoate directly to 2-methylbutanoyl-CoA [40]. Such an enzyme belongs to the 2-oxoacid oxidoreductase family, which is known to oxidatively decarboxylate different 2-oxoacids into their CoA derivatives. Only two members of this family were identified and quantified in our data: the 2-oxoglutarate synthase alpha (Rru_A2721) and beta (Rru_A2722) subunits, which were both downregulated, and the (indole) pyruvate oxidoreductase alpha (Rru_A1978) and beta (Rru_A1977) subunits, which were significantly upregulated under butyrate growth conditions compared to the control succinate growth conditions. While those enzymes seemed to be specific toward defined oxacids [41–43], the InterPro database [18] identified a “pyruvate/ketoisovalerate oxidoreductase” domain (IPR019752) in Rru_A1977. Ketoisovalerate oxidoreductase (Vor) has been poorly studied and are currently only described in archaea [41, 44]. The sequences of VorA and VorB of *Methanothermobacter marburgensis* were blasted against the *Rs. rubrum* genome, and Rru_A2721/2722 and Rru_A1977/78 were identified as the best and second hits, respectively. Rru_A1977 and Rru_A1978 presented 27 and 23% identities with VorA and VorB, respectively. We proposed here that the (indole) pyruvate oxidoreductases alpha (Rru_A1978) and beta (Rru_A1977) subunits might be involved in the conversion of 3-methyl-2-oxopentanoate directly into 2-methylbutanoyl-CoA as the first step of the isoleucine degradation pathway; however, this requires further investigations that fall out of the scope of this paper.

Regardless of the enzyme responsible for the conversion of 3-methyl-2-oxopentanoate into 2-methylbutanoyl-CoA, our observation that ILV can serve as carbon source in *Rs. rubrum*, suggests that the ILV degradation pathway can effectively be used for the photoheterotrophic assimilation of carbon in this strain.

Based on all these considerations, we proposed here that part of the butyrate could be assimilated through the use of enzymes involved in the biosynthesis and degradation pathways of L-isoleucine converting acetyl-CoA into propionyl-CoA and constituting a new assimilation pathway, the methylbutanoyl-CoA (MBC) pathway.

### Enzymes of the EMC and MBC pathways are not sequentially regulated along the growth curve

The two pathways (EMC and MBC) suggested to be involved in the photoassimilation of butyrate differ in their global balance. Starting from butyrate, the balance of the EMC pathway to produce malate corresponds to 1butyrate + 4/3CO_2_ + 3H_2_O + 1ATP + 5/3ETF(ox) + 2/3NADPH + 2/3H^+^ + 2/3FAD + 1/3 NAD^+^ = 4/3malate + 1ADP + P_i_ + 5/3ETF(red) + 2/3NADP^+^ + 2/3 FADH_2_ + 1/2NADH + 1/3H^+^. The carbon/redox balance of the MBC pathway is as follows: 1butyrate + 1 ATP + 5H_2_O + 2ETF(ox) + 2Fd(red) + 2NAD^+^ + 1FAD = 1malate + 1ADP + P_i_ + 2ETF(red) + 2NADH+ 2H^+^ + 1FADH_2_ + 2 Fd (ox). The MBC pathway produces twice as much reduced cofactor per mole of assimilated butyrate which could seem completely unfavorable under photoheterotrophic conditions where redox balance is a major issue [9]. On the other hand, the MBC pathway could lower the dependency of the assimilation of butyrate to the presence of bicarbonate. This could represent an advantage in natural environment or biotechnological application where bicarbonate concentration could be lowered.

We performed a kinetic proteomic analysis to try to determine if these two pathways could be sequentially used for butyrate assimilation. This kinetic proteomic analysis first allowed us to better understand the activation of butyrate. Indeed, our data showed a clear correlation between Rru_A1927, annotated as an acetyl-CoA hydrolase, and the presence of butyrate. The enzyme remained at the same level of abundance and dropped only when butyrate was totally consumed (fold change T4/T3, 0.5; *P*-value, 3.0e-8). On the other hand, the expected butyryl-CoA:acetate CoA transferase, Rru_A1471, which had a lower abundance in the first proteomic analysis was not identified here. These results tend to show that the activation of butyrate is probably entirely mediated by Rru_A1927.

Most of the enzymes involved in the EMC pathway exhibited the similar abundance profile: the expression levels during the exponential phase remained constant while the highest abundances were systematically observed in T4 when butyrate was consumed. This observation appears quite logical according to the hypothesis that the EMC pathway serves as a central carbon metabolism reentry pathway for the depolymerization products of polyhydroxybutyrate, which are mobilized when the carbon source in the medium is totally consumed. The enzymes involved in the remobilization of PHB were all upregulated during the last stage of growth (T4). Interestingly, at this moment of growth, the intracellular PHB depolymerase PhaZ2 was strongly downregulated, while the periplasmic depolymerase PhaZ1 was upregulated. On the other hand, phasin (Rru_A2817) was also strongly downregulated between T3 and T4, the abundance of which dropped to minimal levels. Assuming that the intracellular PHB granules were completely consumed between T3 and T4 (resulting in the lowered abundances in phasins and PhaZ2), PhaZ1 is probably involved in the depolymerization of native PHB granules released in the culture medium through cell lysis as already hypothesized by Sznajder et al. [45].

No significant variations were observed in the abundance of the enzymes involved in the ILV biosynthesis pathway. A very slight and continuous downward trend was observed between T0 and T4, but the fold changes were not biologically relevant (fold changes higher than 0.8). Interestingly, the upregulated transaminase Rru_A2223 exhibited a similar profile with a more significant decrease during the exponential phase (fold change T4/T0: 0.6; *P*-value: 4e-8). The other transaminase, Rru_A0508, showed the opposite trend and was upregulated during the stationary phase. Nevertheless, the abundance remained far lower than that of Rru_A2223.

Concerning the L-isoleucine degradation pathway, the enzymes of the *ivd* cluster showed a similar abundance profile all along the growth curve. On the other hand, LiuR showed a maximum of abundance at the end of the growth phase and an important decrease of abundance at the onset of the stationary phase. Rru_A1942 (LiuC) and Rru_A1944 (unknown function) presented an increased abundance when stationary phase started. In contrast, the two enzymes expected to connect the ILV biosynthesis and L-isoleucine degradation pathways, Rru_A1977 and Rru_A1978, were observed with a clear upregulation exclusively during the butyrate assimilation phase (T1 to T3) followed by a strong downregulation once butyrate is consumed, reinforcing the likelihood of their involvement in butyrate assimilation.

Although our kinetic proteomic approach allowed to highlight a few trends, we could not observe a clear sequential expression of the enzymes of the EMC and MBC pathways that seem therefore to be simultaneously used at least during the analyzed growth period.

### (S)-3-hydroxybutyryl-CoA seems to be the intermediate in conversion of butyrate to acetyl-CoA

Data from the mutant fitness assay revealed that the mutations knocking out the gene Rru_A2964, coding for the enzyme that catalyzes the interconversion of crotonyl-CoA and (R)-3-hydroxybutyryl-CoA, appeared to be beneficial in butyrate growth conditions, meaning that this gene has a deleterious effect on the growth of *Rs. rubrum* when butyrate is the carbon source (Fig. 6). Conversely, the disruption of Rru_A3079 appeared to impede growth on butyrate. This result supports our hypothesis of a preferred route using the (S)-isomer of 3-hydroxybutyryl-CoA for the synthesis of acetyl-CoA under butyrate growth conditions.

### EMC pathway is strictly required for butyrate assimilation

On the other hand, almost all the enzymes of the EMC pathway as well as enzymes involved in the conversion of propionyl-CoA to succinyl-CoA are strongly required for the optimal growth of *Rs. rubrum* on butyrate (Fig. 6). The fitness of strains harboring mutations in Rru_A1201 and Rru_A0217 was also strongly lowered, while proteomic analysis did not show upregulation of these proteins under butyrate conditions. Only 2 genes were missing from our data: ethylmalonyl-CoA/methylmalonyl-CoA epimerase (Rru_A1572), which was excluded from the dataset due to gene length criteria [12], and malyl-CoA thioesterase (Rru_A1200), which was not taken into account because fitness measurements could only be obtained for 2 of the 3 replicates. Based on the mutant fitness assay and proteomic analysis results, the EMC pathway might be considered as a key assimilation pathway for butyrate in *Rs. rubrum*. Nevertheless, based on the transposon library mutant fitness assay, crotonyl-CoA carboxylase/reductase (Rru_A3063), the key enzyme of this pathway, does not appear to be essential in the butyrate assimilation. In the study of the assimilation of acetate, the RB-TnSeq library clearly demonstrated that *ccr* was also essential for growth in medium supplemented with acetate. Data obtained with our (∆*ccr*::Km^R^) mutant confirmed the mutant library observation that ccr was not essential for growth in butyrate conditions. This observation, which contrasted with the essentiality of the other members of the EMC pathway, may suggest the use of an alternative route from butyryl-CoA to ethymalonyl-CoA bypassing the crotonyl-CoA carboxylase/reductase. In 1968, Olsen and Merrick demonstrated that propionyl-CoA carboxylase, identified here as Rru_A0052 and Rru_A0053, also catalyzed the carboxylation of butyryl-CoA, but produced ethylmalonyl-CoA at much lower rate (358 μmole/h.mg proteins vs. 32.2 μmole/h.mg proteins for propionyl-CoA and butyryl-CoA respectively). The ubiquitous activity of propionyl-CoA carboxylase against butyryl-CoA could explain why the EMC pathway is essential for butyrate assimilation, while its key enzyme, CCR, is not.

### MBC pathway is not strictly required for growth with butyrate and ILV biosynthesis and degradation could be decoupled

The ILV biosynthesis pathway or at least the conversion of pyruvate to 3-methyl-2-oxopentanoate appears to be essential (Fig. 6). This requirement may be related to adaptation to minimal medium since negative fitness values are also observed for succinate. Nevertheless, the overall fitness values appeared to be lower when the mutant library was grown on butyrate. This observation reveals that if ILV biosynthesis is required in both succinate and butyrate conditions, the growth defect is bigger in butyrate than in succinate medium, suggesting a specific involvement of this pathway in the adaptation to butyrate.

On the other hand, genes involved in the degradation of ILV and belonging to the *ivd* and *liu* pathways were not detected as being essential for growth in butyrate conditions (Fig. 6). This result could reflect a certain degree of complementarity between the *ivd* and *liu* gene clusters or that MBC is only an accessory pathway for butyrate assimilation. An alternative explanation of the observed mutant fitness for the genes involved in the MBC pathway could be linked with the decoupling of the two parts of the MBC pathway, the ILV biosynthesis and ILV degradation pathways. The ILV biosynthesis genes seem indeed to be involved in the adaptation of *Rs. rubrum* to butyrate photoassimilation and an increased isoleucine production has been demonstrated in the early phase of the butyrate assimilation (Fig. 4). This isoleucine production could be linked with redox balancing since the isoleucine production consumes reducing power. In a recent study of the adaptation of *Rs. rubrum* to sudden increase in light intensity, we proposed a transient decoupling of the biosynthesis of ILV, consuming excess of reducing power, from the degradation of these ILV, releasing stored nutrient and reducing power. The results of the mutant fitness are compatible with such a hypothesis since mutations affecting the ILV biosynthesis exhibit higher impact on mutant fitness in butyrate condition than mutations affecting the ILV degradation, the latter only affecting the growth during the late carbon depleted phase of the growth curve. The regulation of the metabolic fluxes through the MBC pathway would definitely require further analyses. However, the results presented here clearly demonstrated that higher production of isoleucine occurs during butyrate photoassimilation, at least during the early phase of the growth, and could be necessary for adaptation to this carbon source.

## Conclusion

Based on proteomic and mutant fitness analyses in this study, we paved the way for a better understanding of butyrate metabolism under photoheterotrophic conditions in *Rs. rubrum*. Both proteomic and mutant fitness data revealed the involvement of the previously described ethylmalonyl-CoA pathway in butyrate assimilation. In addition to this main assimilation pathway, the ILV biosynthesis was also observed as potentially involved in butyrate assimilation. The production of isoleucine was shown to increase in butyrate condition, at least during the early phase of growth, which might suggest involvement of isoleucine production in redox balancing. Enzymes from the ILV degradation were also upregulated under butyrate conditions which suggests that produced isoleucine can be further assimilated when redox balance is not an issue anymore. *Rs. rubrum* has been shown to be able to use ILV has a carbon source. The conversion of butyrate to isoleucine, for redox balancing, and the further reassimilation of isoleucine could constitute a new pathway of butyrate assimilation that we here call the MBC pathway. The measurement of actual fluxes through the MBC pathway, its use as a redox balancing mechanism and its regulation would require further analyses.

## Methods

### Bacterial strain, medium composition and cultivation conditions

*Rs. rubrum* S1H (ATCC 25903) was used in this study using medium and culture conditions already described [13]. This strain has a higher threonine deaminase content than the parent strain S1 (ATCC1170), making it less susceptible to inhibition by the presence of exogenous L-threonine [46]. The culture medium was supplemented with either butyrate (31 mM) or succinate (31 mM; control) as the carbon source. Bicarbonate was supplemented during growth with the addition of NaHCO_3_ filtered solution (70 mM or 120 mM, depending on the experiment). For comparison of PHA production through electron microscopy, cells were also obtained from acetate-supplemented medium (62 mM acetate, 3 mM bicarbonate). The cultivation experiments performed with mutant strains under photoheterotrophic conditions were performed with 3 mM bicarbonate in the culture medium.

Anaerobic phototrophic conditions were obtained by purging upper gas phase with pure nitrogen before hermetically sealing the flask. Cultures were inoculated at a starting OD_680nm_ between 0.5 and 0.7, light was supplied by halogen lamps (Sencys; 10 W; 100 lm; 2650 K) at an intensity of 50 μmole.m^− 2^.sec^− 1^. Under dark aerobic conditions, cultures were inoculated at a starting OD_680nm_ of 0.1, wrapped in aluminum foil and incubated at 30 °C with shaking at 150 rpm. Three monoclonal biological replicates were performed for each culture condition, except for the cultivation experiments with mutant strains that were performed on five monoclonal biological replicates. The growth was monitored by measuring the optical density at 680 nm using a 1-cm-path-length cuvette and a Thermo Scientific Helios Zeta spectrophotometer. When the OD was higher than 1.0, the samples were diluted, and the measured OD values were corrected for the dilution.

### Proteomic analysis using mass spectrometry

Quantitative proteomic analyses were performed on protein extracts of *Rs. rubrum* S1H cultivated in medium supplemented with butyrate or succinate. For each experiment, cells from 5 biological replicates were used to extract proteins and obtain tryptic peptides following already described procedure [13]. A SWATH based proteomic analysis was performed using an UHPLC-HRMS/MS platform (Eksigent 2D ultra & AB Sciex TripleTOF™ 5600) with same acquisition parameters as previously reported [13] with two micrograms of peptides. The used reference spectral library resulted from the DDA analysis of proteomes obtained from *Rs. rubrum* S1H grown on acetate, succinate and butyrate containing medium using the *Rs. rubrum* ATCC11170 UniProt entries (UPID: UP000001929; May 2013) as reference sequence database.

SWATH wiff files were processed using the AB Sciex PeakView 2.1 software and the SWATH™ Acquisition MicroApp considering up to 6 peptides (with at least 99% confidence) and 6 transitions per peptide. The XIC peak area was extracted and exported in AB Sciex MarkerView™ 1.2 software for normalization using Total Area Sums. A principal component analysis (PCA) was routinely performed on normalized data to eventually highlight trends across groups of samples. The significance of the relative abundance (fold change; FC) observed between our experimental conditions was determined through Student’s *t*-test with a statistical threshold set at 5% (*P*-value < 0.05).

### Monitoring of volatile fatty acids consumption

VFA consumption was monitored through culture supernatants obtained by centrifugation at 16,000 g for 10 min at 4 °C and stored at − 20 °C before analysis. One hundred microliters of culture supernatant were analyzed by HPLC-refractometry (Waters 2695 Separation Module; Waters 2414 Refractive Index Detector). The separation was realized in isocratic mode using a Shodex Sugar SH1011 column (300 mm × 8 mm) with 5 mM H_2_SO_4_ as the mobile phase (flow rate: 1 ml/min.). The detection was performed by refractometry at 210 nm. The amounts of succinate, butyrate and acetate were determined by integrating their specific peaks (9, 11, 16 min RT, respectively) and compared to standard curves.

### Measurement of amino acids abundances in the biomass

The pellets issued from the centrifugation of 500 μL of culture of Rs. rubrum in succinate or butyrate condition were snap frozen using liquid nitrogen, freeze-dried and stored at − 20 °C until the extraction. Briefly, the pellets were resuspended using 1.5 mL of a methanol:chloroform solution (1:2, v:v). Five freeze/thaw cycles were applied to the solution and 400 μL of distilled H_2_O were added. After centrifugation (5000 g, 4 °C), the aqueous upper phase was collected and submitted to SpeedVac. Dried samples were solubilized in loading buffer (acetonitrile 3% (v/v), formic acid 1% (v/v)). The metabolites were analyzed using an eksigent LC 425 system coupled to a Q-TRAP6500+ (Sciex) used in multiple reaction monitoring (MRM) mode. Peptides were separated on a C18 YMC-Triat 0.3x150mm column operated at a flow rate of 5 μl/min in isocratic mode (3% acetonitrile (v/v), formic acid 1% (v/v)) for 5 min followed by an acetonitrile gradient from 3 to 55% in 3 min. Following transitions were used to quantify the following amino acids: Lysine 147/130; Valine 118/72 Arginine 175/116 and isoleucine 132/69. In order to avoid extraction bias between the samples, isoleucine abundance was expressed as the ratio of the area under the curve for its specific transition on the area under the curve of the specific transition of each of the other amino acids.

### Transmission electron microscopy

Cells were grown to the mid-exponential growth phase (OD_680nm_ around 1), harvested by centrifugation at 16,000 g for 10 min at 4 °C, washed with a cacodylate buffer (0.1 M cacodylate, 8 mM NaCl, 0.05% (w/v) ruthenium red, pH 7.8) and immediately fixed with 3% (v/v) glutaraldehyde in cacodylate buffer for 2 h at 4 °C. The samples were rinsed with cacodylate buffer (2 × 10 min.), postfixed for 2 h with 1% (w/v) OsO_4_ cacodylate buffer at room temperature and washed again. Subsequent dehydration steps were conducted in a graded ethanol series up to 100%. Dehydrated samples were finally embedded in Spurr resin (TAAB Laboratories Equipment, Berks, England). Ultrathin slices (70–90 μm) were obtained with a Leica Ultracut UCT ultramicrotome and stained with uranyl acetate and lead citrate. Prepared slices were observed with a Zeiss LEO 906E transmission electron microscope operated at 60 kV, and TEM images were acquired with analySIS (Soft Imaging System, Switzerland) software.

### Genome-wide mutant fitness assays on *Rs. rubrum* S1H transposon mutant library

The mutant library was produced following the protocol described by Wetmore et al. (2015) [12], which is briefly described in the Supporting Information.

The mutant fitness assays were performed as described previously [13] using three different glycerol stocks from the mutant library (triplicate). Only gene for which a difference of fitness was observed between succinate and butyrate conditions (as determine through unpaired Student’s *t*-test with a statistical threshold set at 5%) were further considered as essential to adapt specifically to butyrate condition. Further details about mutant fitness experiments are given in Supporting Information.

## Supplementary information

**Additional file 1:Table S1.** Differential protein abundance in butyrate- vs succinate-grown *Rhodospirillum rubrum* S1H (Complete Dataset). **Table S2.** Sequence comparison for ILV degradation pathway in *Rs. rubrum*, based on *Shewanella oneidensis* ivd operon. **Table S3.** Differential protein abundance in butyrate-grown *Rhodospirillum rubrum* S1H during different growth phases (Complete Dataset). **Table S4.** Fitness profiling of and succinate- and butyrate-grown *Rhodospirillum rubrum* S1H (Complete Dataset).

**Additional file 2: Fig. S1.** Targeted mutagenesis of the crotonyl-CoA carboxylase/reductase (*ccr* - Rru_A3063). Scaled diagram of the *ccr* gene region both in the wild type and ∆*ccr*::Km^R^ strain. “H.R.1” and “H.R.2” are the homologous regions used for the final double homologous recombination between the genomic DNA and cloning vector, and “Km^R^ Cass.” is the kanamycin antibiotic resistance cassette. **Fig. S2.** MRM based quantification of abundance of Rru_A3062 (A), Rru_A3063 (B) and Rru_A3064 (C) wt and ∆*ccr*::Km^R^ strain. We use 5, 3 and 7 peptides to quantify Rru_A3062, Rru_A3063 and Rru_A3064, respectively, based on 6 transitions for each peptide. Five biological replicate cultures of wt (orange bar) and ∆*ccr*::Km^R^ strains (blue) were grown in butyrate containing medium and harvested in mid-exponential phase. **Fig. S3.** Growth of *Rs. rubrum* S1H in medium supplemented with succinate or acetate and a mixture of ILV. *Rs. rubrum* was cultivate under anaerobic photoheterotrophic conditions in medium containing 31 mM of succinate (A) or acetate (B) and 3 mM of bicarbonate (dashed lines). The medium was also eventually supplemented with an equimolar (10 mM each) mixture of ILV (solid lines). The growth was monitored by measuring OD_680nm_. *n* = 5. **Table S5.** Oligonucleotides used for targeted mutagenesis. **Table S6.** MRM transition used to monitor abundance of protein expressed by gene close to Rru_A3063.

## Data Availability

Complete datasets of proteomic and functional genomic are available as supporting tables of this manuscript. All raw proteomic data and BarSeq data (functional genomic) can be obtained by simple request to the corresponding author.

## Abbreviations

PNSB: Purple non sulfur bacteria
VFA: Volatile fatty acid
MELiSSA: Micro-Ecological Life Support System Alternative
Icl-: Isocitrate lyase-lacking
EMC: Ethylmalonyl-CoA
ILV: Isoleucine, leucine and valine
IBC: Isobutyryl-CoA
MRM: Multiple reaction monitoring
PHB: Poly (3-hydroxybutyrate)
SWATH: Sequential window acquisition of all theoretical mass spectra
XIC: Extracted ion chromatogram
