# Supplementary material for: New perspectives on butyrate assimilation in Rhodospirillum rubrum S1H under photoheterotrophic conditions
Source: BMC Microbiol. 2020 May 20;20:126. doi: 10.1186/s12866-020-01814-7 (PMC7238569; doi:10.1186/s12866-020-01814-7)
Supplement: Supplementary file 2 — Additional file 2: Fig. S1. Targeted mutagenesis of the crotonyl-CoA carboxylase/reductase (ccr - Rru_A3063). Scaled diagram of the ccr gene region both in the wild type and ∆ccr::KmR strain. “H.R.1” and “H.R.2” are the homologous regions used for the final double homologous recombination between the genomic DNA and cloning vector, and “KmR Cass.” is the kanamycin antibiotic resistance cassette. Fig. S2. MRM based quantification of abundance of Rru_A3062 (A), Rru_A3063 (B) and Rru_A3064 (C) wt and ∆ccr::KmR strain. We use 5, 3 and 7 peptides to quantify Rru_A3062, Rru_A3063 and Rru_A3064, respectively, based on 6 transitions for each peptide. Five biological replicate cultures of wt (orange bar) and ∆ccr::KmR strains (blue) were grown in butyrate containing medium and harvested in mid-exponential phase. Fig. S3. Growth of Rs. rubrum S1H in medium supplemented with succinate or acetate and a mixture of ILV. Rs. rubrum was cultivate under anaerobic photoheterotrophic conditions in medium containing 31 mM of succinate (A) or acetate (B) and 3 mM of bicarbonate (dashed lines). The medium was also eventually supplemented with an equimolar (10 mM each) mixture of ILV (solid lines). The growth was monitored by measuring OD680nm. n = 5. Table S5. Oligonucleotides used for targeted mutagenesis. Table S6. MRM transition used to monitor abundance of protein expressed by gene close to Rru_A3063. [file 12866_2020_1814_MOESM2_ESM.docx]

**Supporting Information**

**Supporting Text**

**Study of polar effect in** ∆*ccr*::Km^R^ **mutant**

In order to quantify changes in expression of neighbor genes potentially occuring due to insertion of the kanamycin cassette and because of the organization of this genomic region (Fig. S1), we decided to quantify abundance of proteins Rru_A3062 and Rru_A3064 using a targeted proteomic approaches called MRM. Butyrate grown samples were considered perfect for this because butyrate trigger expression of Rru_A3062, Rru_A3063 and Rru_A3064 as demonstrated by our proteomic analysis but the ∆*ccr*::Km^R^ mutant is anyway able to grow in this conditions. Sample were prepared as described for proteomic analysis and analyzed on a QTRAP6500+ coupled to an eksigent 425 operated in micromode (5µl/min). 2µg of peptides were separated on a YMC triart C18 column (0.3x150mm) along a 2-35% acetonitrile 20 minutes gradient. Proteins were relatively quantified between wt and mutant samples through monitoring of 7, 5 and 4 peptides for rru_A3064, Rru_A3063 and Rru_A3062 respectivelly. Each peptide was quantified using at least 6 transition, the full transition list is provided in Table S6 here under.

All three proteins could be accurately quantified based on these transitions. Fig. S2 shows that Rru_A3063 is absent from the mutant strains as expected. Regarding influence of the insertion of the kanamycin cassette, if no significant changes can be observed for Rru_A3062, it is clear that expression of Rru_A3064, if still occurring is affected by the insertion. We calculate that abundance of Rru_A3064 is decrease by 2.5 time in mutant strains as compared with the wt. Anyway as Rru_A3063 and rru_A3064 belong to the same metabolic pathway this polar effect is not expected to have major impact on the results.

**Experimental procedure of the mutant fitness assay**

The mutant library used for this experiment was produced in rich medium under aerobic conditions without light, the least selective growth conditions for our strain. The library was first amplified in rich SMN medium under photoheterotrophic conditions before being exposed to minimal medium containing succinate or butyrate. The comparison of the fitness of the library grown in the presence of butyrate to the fitness profile obtained using succinate as a control carbon source allows to discriminate genes specifically involved in butyrate photoassimilation from genes involved in the adaptation to minimal medium.

The fitness value of a given gene is determined as the average of the fitness values measured for all the strains carrying transposon insertions in this specific gene (average number of different strains per gene in our *Rs. rubrum* mutant library: 27). Each strain fitness value is defined as the log_2_ of the ratio between the strain abundance after 5 generations and its abundance at the beginning of the experiment in the relevant conditions (Wetmore et al. 2015).

The robustness of the data was ensured at different levels. The experiment was conducted as three independent replicates consisting of the independent amplification of different stocks of the library. The minimum read counts per strain was set at 10 to ensure the acquisition of high confidence data. Only genes for which the fitness value was lower than -0.5 in the butyrate condition and significantly different from the fitness value in the succinate condition (P-value < 0.05; unpaired t-test) were considered as involved in butyrate metabolism.

**Supporting Figures**

**Fig. S1. Targeted mutagenesis of the crotonyl-CoA carboxylase/reductase (*ccr* - Rru_A3063).** Scaled diagram of the *ccr* gene region both in the wild type and ∆*ccr*::Km^R^ strain. “H.R.1” and “H.R.2” are the homologous regions used for the final double homologous recombination between the genomic DNA and cloning vector, and “Km^R^ Cass.” is the kanamycin antibiotic resistance cassette.

**Fig. S1**

**
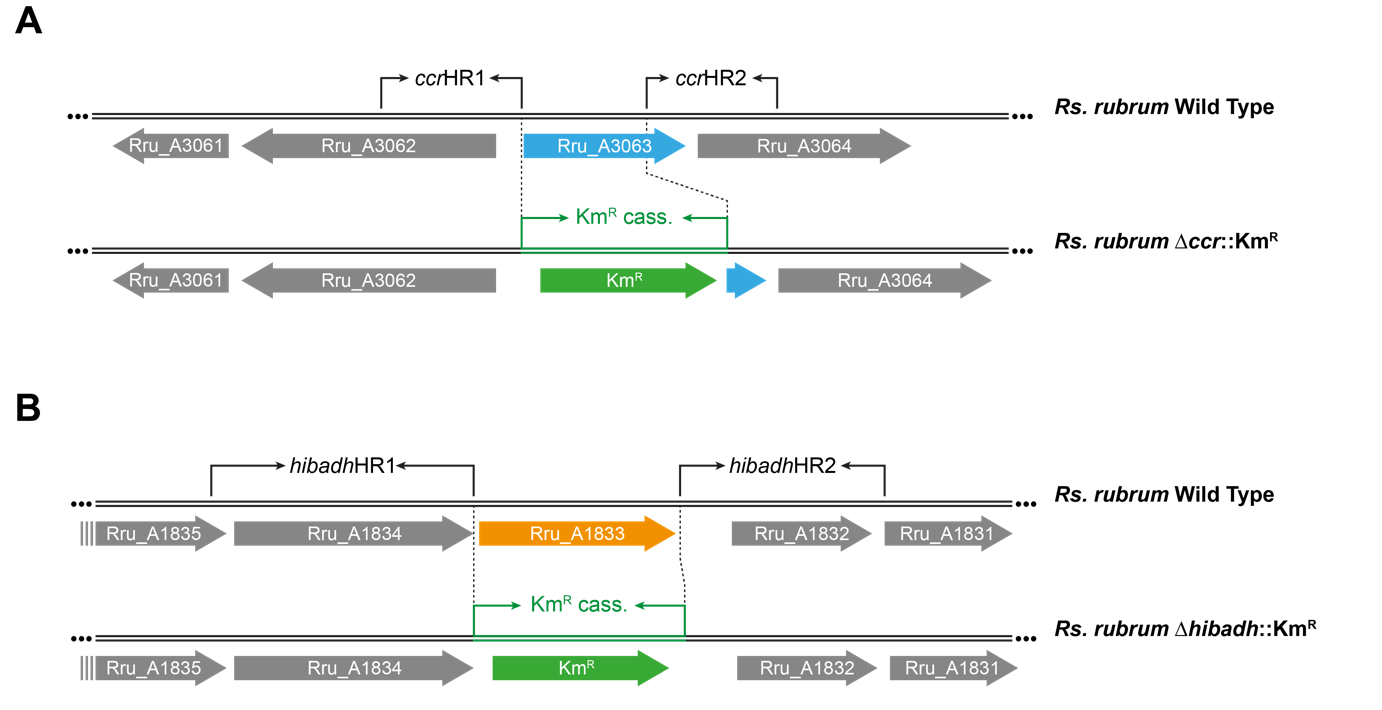
**

**Fig. S2. MRM based quantification of abundance of Rru_A3062 (A), Rru_A3063 (B) and Rru_A3064 (C) wt and ∆*ccr*::Km^R^ strain.** We use 5, 3 and 7 peptides to quantify Rru_A3062, Rru_A3063 and Rru_A3064, respectively, based on 6 transitions for each peptide. Five biological replicate cultures of wt (orange bar) and ∆*ccr*::Km^R^ strains (blue) were grown in butyrate containing medium and harvested in mid-exponential phase.

**Fig. S2**


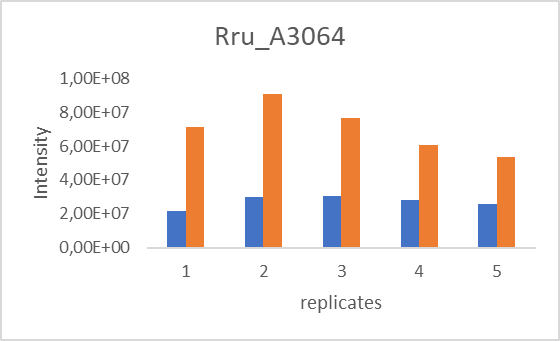

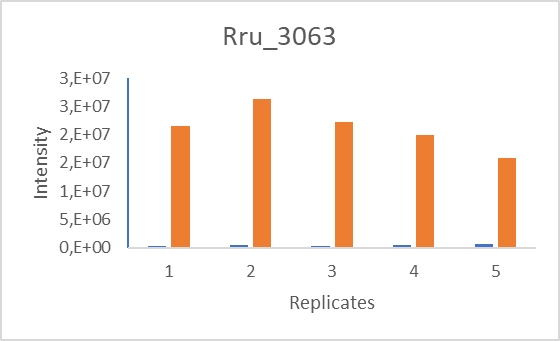

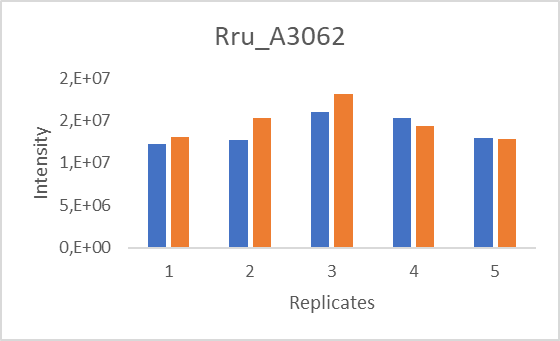


**Fig. S3. Growth of *Rs. rubrum* S1H in medium supplemented with succinate or acetate and a mixture of ILV.** *Rs. rubrum* was cultivate under anaerobic photoheterotrophic conditions in medium containing 31 mM of succinate (A) or acetate (B) and 3mM of bicarbonate (dashed lines). The medium was also eventually supplemented with an equimolar (10 mM each) mixture of ILV (solid lines). The growth was monitored by measuring OD_680nm_. n=5.

**
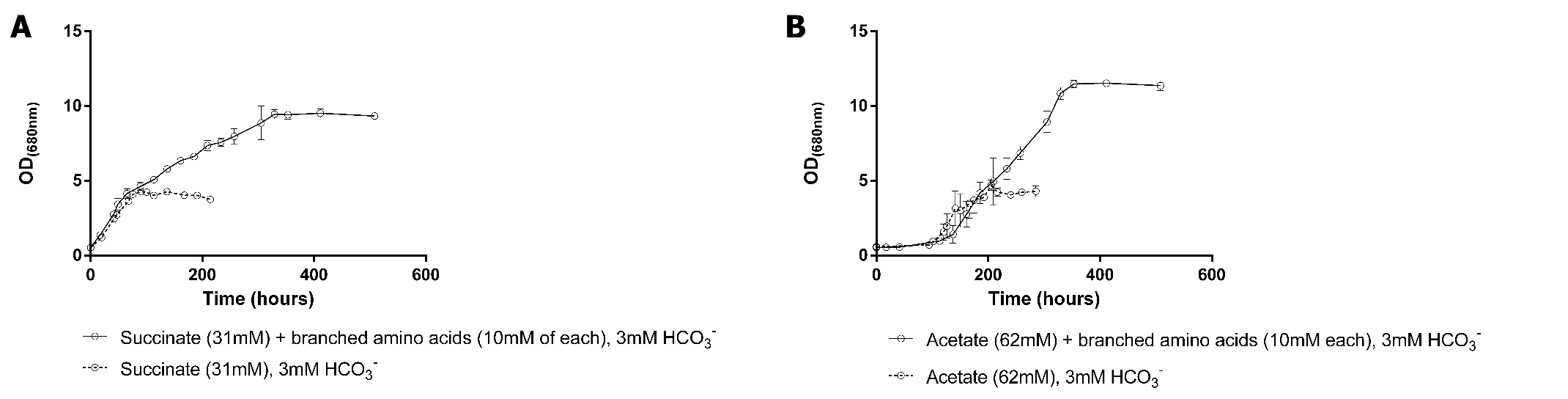
**

**Supporting Tables**

| **Table S5. Oligonucleotides used for targeted mutagenesis.** | | | |
| --- | --- | --- | --- |
| **Source** | **Name^a^** | **Sequence, 5’-3’ ^b, c, d^** | **Application** |
| ***E. coli* K12 MC1061** | | | |
| pACY177 | Km_Fw | TCTCTGATGTTACATTGCAC | Mutagenesis |
| pACY177 | Km_Rv | CAGCGTAATGCTCTGC | Mutagenesis |
| ***E. coli* K12 MA8** | | | |
| pKNOCK-Gm | pKNOCK_Fw | CATAAGCCTGTTCGGT | Mutagenesis |
| pKNOCK-Gm | pKNOCK_Fw | CGGGGGATCCACTAG | Mutagenesis |
| ***Rhodospirillum rubrum* S1H** | | | |
| Genomic DNA | ccrHR1_Fw | AGAACTAGTGGATCCGGCGTTCTTTGACAAGACCA | Mutagenesis |
| Genomic DNA | ccrHR1_Rv | AATGTAACATCAGAGAGTTGAAACCTCTAGGCCGCT | Mutagenesis |
| Genomic DNA | ccrHR2_Fw | GCAGAGCATTACGCTGAAGAGGTGCGCAAGTTCG | Mutagenesis |
| Genomic DNA | ccrHR2_Rv | ACCGAACAGGCTTATGTCGCTCAGGTGCCATTC | Mutagenesis |
| Genomic DNA | hibadhHR1_Fw | AGAACTAGTGGATCCTCACCGGATCTTGGAAGGC | Mutagenesis |
| Genomic DNA | hibadhHR1_Rv | AATGTAACATCAGAGATTAAGGGTCGCGAAACTCCA | Mutagenesis |
| Genomic DNA | hibadhHR2_Fw | GCAGAGCATTACGCTGGTGGAACGCGACAACACCA | Mutagenesis |
| Genomic DNA | hibadhHR2_Rv | ACCGAACAGGCTTATGTTGGCTGACCTCGACCTCAG | Mutagenesis |
| Genomic DNA | Ccr_Fw | GTCAACTACAACGGGATCTGGG | Mutant Screening |
| Genomic DNA | Ccr_Rv | GAAATCGCGCTTGGTCTCATC | Mutant Screening |
| Genomic DNA | hibadh_Fw | CCTTAACTTTCATGGTTGGCGG | Mutant Screening |
| Genomic DNA | hibadh_Rv | GCCTTGGAACTGATGTCGAAC | Mutant Screening |
| ^a^ Fw, forward primer; Rv, reverse primer  ^b^ Nucleotide sequences based on the sequences of the pACY117 plasmid (GenBank: X06402.1), the pKNOCK-Gm plasmid and the Rhodospirillum rubrum str. ATCC11170 (NCBI Reference sequence: NC_007643.1)  ^c^ Underligned sequences are end-terminal homology sequences required for GeneArt Seamless cloning  All the oligonucleotides were designed using Primer3Plus v2.4.0, checked for specificity using Snapgene Viewer software (GSL Biotech) and for the formation of dimers and secondary structures using the Multiple Primer Analyzer webtool (ThermoFisher Scientific), and were obtained from Eurogentec S.A. | | | |

**Table S6. MRM transition used to monitor abundance of protein expressed by gene close to Rru_A3063.**

| **Q1** | **Q3** | **protein and peptide reference** | **Collision energy** |
| --- | --- | --- | --- |
| 789,913 | 1265,62 | tr\|Q2RPT6\|Q2RPT6_RHORT.SLILPDLLYTC[CAM]DR.+2y10.light | 41,5 |
| 789,913 | 1152,535 | tr\|Q2RPT6\|Q2RPT6_RHORT.SLILPDLLYTC[CAM]DR.+2y9.light | 41,5 |
| 789,913 | 940,456 | tr\|Q2RPT6\|Q2RPT6_RHORT.SLILPDLLYTC[CAM]DR.+2y7.light | 41,5 |
| 789,913 | 551,224 | tr\|Q2RPT6\|Q2RPT6_RHORT.SLILPDLLYTC[CAM]DR.+2y4.light | 41,5 |
| 789,913 | 201,123 | tr\|Q2RPT6\|Q2RPT6_RHORT.SLILPDLLYTC[CAM]DR.+2b2.light | 41,5 |
| 789,913 | 314,207 | tr\|Q2RPT6\|Q2RPT6_RHORT.SLILPDLLYTC[CAM]DR.+2b3.light | 41,5 |
| 510,28 | 906,468 | tr\|Q2RPT6\|Q2RPT6_RHORT.LADFGIEVR.+2y8.light | 27,5 |
| 510,28 | 835,431 | tr\|Q2RPT6\|Q2RPT6_RHORT.LADFGIEVR.+2y7.light | 27,5 |
| 510,28 | 720,404 | tr\|Q2RPT6\|Q2RPT6_RHORT.LADFGIEVR.+2y6.light | 27,5 |
| 510,28 | 573,336 | tr\|Q2RPT6\|Q2RPT6_RHORT.LADFGIEVR.+2y5.light | 27,5 |
| 510,28 | 274,187 | tr\|Q2RPT6\|Q2RPT6_RHORT.LADFGIEVR.+2y2.light | 27,5 |
| 510,28 | 300,155 | tr\|Q2RPT6\|Q2RPT6_RHORT.LADFGIEVR.+2b3.light | 27,5 |
| 688,401 | 1092,605 | tr\|Q2RPT6\|Q2RPT6_RHORT.GLLQTPYTTLLR.+2y9.light | 36,4 |
| 688,401 | 964,546 | tr\|Q2RPT6\|Q2RPT6_RHORT.GLLQTPYTTLLR.+2y8.light | 36,4 |
| 688,401 | 863,499 | tr\|Q2RPT6\|Q2RPT6_RHORT.GLLQTPYTTLLR.+2y7.light | 36,4 |
| 688,401 | 603,382 | tr\|Q2RPT6\|Q2RPT6_RHORT.GLLQTPYTTLLR.+2y5.light | 36,4 |
| 688,401 | 284,197 | tr\|Q2RPT6\|Q2RPT6_RHORT.GLLQTPYTTLLR.+2b3.light | 36,4 |
| 688,401 | 513,303 | tr\|Q2RPT6\|Q2RPT6_RHORT.GLLQTPYTTLLR.+2b5.light | 36,4 |
| 841,774 | 1342,721 | tr\|Q2RPT6\|Q2RPT6_RHORT.IAALIADGHFGDPGLEDETLTLVR.+3y12.light | 40,6 |
| 841,774 | 1075,563 | tr\|Q2RPT6\|Q2RPT6_RHORT.IAALIADGHFGDPGLEDETLTLVR.+3y9.light | 40,6 |
| 841,774 | 946,52 | tr\|Q2RPT6\|Q2RPT6_RHORT.IAALIADGHFGDPGLEDETLTLVR.+3y8.light | 40,6 |
| 841,774 | 702,451 | tr\|Q2RPT6\|Q2RPT6_RHORT.IAALIADGHFGDPGLEDETLTLVR.+3y6.light | 40,6 |
| 841,774 | 185,128 | tr\|Q2RPT6\|Q2RPT6_RHORT.IAALIADGHFGDPGLEDETLTLVR.+3b2.light | 40,6 |
| 841,774 | 256,166 | tr\|Q2RPT6\|Q2RPT6_RHORT.IAALIADGHFGDPGLEDETLTLVR.+3b3.light | 40,6 |
| 547,304 | 859,5 | tr\|Q2RPT6\|Q2RPT6_RHORT.AYIGVGSLGTR.+2y9.light | 29,4 |
| 547,304 | 746,416 | tr\|Q2RPT6\|Q2RPT6_RHORT.AYIGVGSLGTR.+2y8.light | 29,4 |
| 547,304 | 689,394 | tr\|Q2RPT6\|Q2RPT6_RHORT.AYIGVGSLGTR.+2y7.light | 29,4 |
| 547,304 | 590,326 | tr\|Q2RPT6\|Q2RPT6_RHORT.AYIGVGSLGTR.+2y6.light | 29,4 |
| 547,304 | 235,108 | tr\|Q2RPT6\|Q2RPT6_RHORT.AYIGVGSLGTR.+2b2.light | 29,4 |
| 547,304 | 405,213 | tr\|Q2RPT6\|Q2RPT6_RHORT.AYIGVGSLGTR.+2b4.light | 29,4 |
| 524,297 | 845,528 | tr\|Q2RPT6\|Q2RPT6_RHORT.SDLMTLLVR.+2y7.light | 28,2 |
| 524,297 | 732,444 | tr\|Q2RPT6\|Q2RPT6_RHORT.SDLMTLLVR.+2y6.light | 28,2 |
| 524,297 | 601,403 | tr\|Q2RPT6\|Q2RPT6_RHORT.SDLMTLLVR.+2y5.light | 28,2 |
| 524,297 | 500,355 | tr\|Q2RPT6\|Q2RPT6_RHORT.SDLMTLLVR.+2y4.light | 28,2 |
| 524,297 | 387,271 | tr\|Q2RPT6\|Q2RPT6_RHORT.SDLMTLLVR.+2y3.light | 28,2 |
| 524,297 | 316,15 | tr\|Q2RPT6\|Q2RPT6_RHORT.SDLMTLLVR.+2b3.light | 28,2 |
| 474,935 | 863,477 | tr\|Q2RPT6\|Q2RPT6_RHORT.VQFGKPLYAFPR.+3y7.light | 23,3 |
| 474,935 | 653,341 | tr\|Q2RPT6\|Q2RPT6_RHORT.VQFGKPLYAFPR.+3y5.light | 23,3 |
| 474,935 | 490,277 | tr\|Q2RPT6\|Q2RPT6_RHORT.VQFGKPLYAFPR.+3y4.light | 23,3 |
| 474,935 | 272,172 | tr\|Q2RPT6\|Q2RPT6_RHORT.VQFGKPLYAFPR.+3y2.light | 23,3 |
| 474,935 | 598,335 | tr\|Q2RPT6\|Q2RPT6_RHORT.VQFGKPLYAFPR.+3y10+2.light | 23,3 |
| 474,935 | 228,134 | tr\|Q2RPT6\|Q2RPT6_RHORT.VQFGKPLYAFPR.+3b2.light | 23,3 |
| 888,98 | 1143,652 | tr\|Q2RPT7\|Q2RPT7_RHORT.DLYELGEIPPLGHVPK.+2y11.light | 46,4 |
| 888,98 | 844,504 | tr\|Q2RPT7\|Q2RPT7_RHORT.DLYELGEIPPLGHVPK.+2y8.light | 46,4 |
| 888,98 | 747,451 | tr\|Q2RPT7\|Q2RPT7_RHORT.DLYELGEIPPLGHVPK.+2y7.light | 46,4 |
| 888,98 | 392,182 | tr\|Q2RPT7\|Q2RPT7_RHORT.DLYELGEIPPLGHVPK.+2b3.light | 46,4 |
| 888,98 | 521,224 | tr\|Q2RPT7\|Q2RPT7_RHORT.DLYELGEIPPLGHVPK.+2b4.light | 46,4 |
| 888,98 | 820,372 | tr\|Q2RPT7\|Q2RPT7_RHORT.DLYELGEIPPLGHVPK.+2b7.light | 46,4 |
| 937,939 | 1355,623 | tr\|Q2RPT7\|Q2RPT7_RHORT.IWGYETPDGSFAQFTR.+2y12.light | 48,9 |
| 937,939 | 1226,58 | tr\|Q2RPT7\|Q2RPT7_RHORT.IWGYETPDGSFAQFTR.+2y11.light | 48,9 |
| 937,939 | 1125,532 | tr\|Q2RPT7\|Q2RPT7_RHORT.IWGYETPDGSFAQFTR.+2y10.light | 48,9 |
| 937,939 | 913,453 | tr\|Q2RPT7\|Q2RPT7_RHORT.IWGYETPDGSFAQFTR.+2y8.light | 48,9 |
| 937,939 | 300,171 | tr\|Q2RPT7\|Q2RPT7_RHORT.IWGYETPDGSFAQFTR.+2b2.light | 48,9 |
| 937,939 | 357,192 | tr\|Q2RPT7\|Q2RPT7_RHORT.IWGYETPDGSFAQFTR.+2b3.light | 48,9 |
| 650,351 | 1072,568 | tr\|Q2RPT7\|Q2RPT7_RHORT.VQSQQVMARPR.+2y9.light | 34,5 |
| 650,351 | 857,477 | tr\|Q2RPT7\|Q2RPT7_RHORT.VQSQQVMARPR.+2y7.light | 34,5 |
| 650,351 | 630,35 | tr\|Q2RPT7\|Q2RPT7_RHORT.VQSQQVMARPR.+2y5.light | 34,5 |
| 650,351 | 536,788 | tr\|Q2RPT7\|Q2RPT7_RHORT.VQSQQVMARPR.+2y9+2.light | 34,5 |
| 650,351 | 228,134 | tr\|Q2RPT7\|Q2RPT7_RHORT.VQSQQVMARPR.+2b2.light | 34,5 |
| 650,351 | 443,225 | tr\|Q2RPT7\|Q2RPT7_RHORT.VQSQQVMARPR.+2b4.light | 34,5 |
| 433,903 | 428,273 | tr\|Q2RPT7\|Q2RPT7_RHORT.VQSQQVMARPR.+3y3.light | 21,4 |
| 433,903 | 272,172 | tr\|Q2RPT7\|Q2RPT7_RHORT.VQSQQVMARPR.+3y2.light | 21,4 |
| 433,903 | 600,817 | tr\|Q2RPT7\|Q2RPT7_RHORT.VQSQQVMARPR.+3y10+2.light | 21,4 |
| 433,903 | 536,788 | tr\|Q2RPT7\|Q2RPT7_RHORT.VQSQQVMARPR.+3y9+2.light | 21,4 |
| 433,903 | 493,272 | tr\|Q2RPT7\|Q2RPT7_RHORT.VQSQQVMARPR.+3y8+2.light | 21,4 |
| 433,903 | 228,134 | tr\|Q2RPT7\|Q2RPT7_RHORT.VQSQQVMARPR.+3b2.light | 21,4 |
| 452,25 | 832,456 | tr\|Q2RPT7\|Q2RPT7_RHORT.AIWDITGK.+2y7.light | 24,6 |
| 452,25 | 719,372 | tr\|Q2RPT7\|Q2RPT7_RHORT.AIWDITGK.+2y6.light | 24,6 |
| 452,25 | 533,293 | tr\|Q2RPT7\|Q2RPT7_RHORT.AIWDITGK.+2y5.light | 24,6 |
| 452,25 | 418,266 | tr\|Q2RPT7\|Q2RPT7_RHORT.AIWDITGK.+2y4.light | 24,6 |
| 452,25 | 305,182 | tr\|Q2RPT7\|Q2RPT7_RHORT.AIWDITGK.+2y3.light | 24,6 |
| 452,25 | 185,128 | tr\|Q2RPT7\|Q2RPT7_RHORT.AIWDITGK.+2b2.light | 24,6 |
| 813,103 | 1157,627 | tr\|Q2RPT7\|Q2RPT7_RHORT.IQGSHFANLLQASQANQLVIER.+3y10.light | 39,2 |
| 813,103 | 942,537 | tr\|Q2RPT7\|Q2RPT7_RHORT.IQGSHFANLLQASQANQLVIER.+3y8.light | 39,2 |
| 813,103 | 871,5 | tr\|Q2RPT7\|Q2RPT7_RHORT.IQGSHFANLLQASQANQLVIER.+3y7.light | 39,2 |
| 813,103 | 417,246 | tr\|Q2RPT7\|Q2RPT7_RHORT.IQGSHFANLLQASQANQLVIER.+3y3.light | 39,2 |
| 813,103 | 968,495 | tr\|Q2RPT7\|Q2RPT7_RHORT.IQGSHFANLLQASQANQLVIER.+3b9.light | 39,2 |
| 813,103 | 1081,579 | tr\|Q2RPT7\|Q2RPT7_RHORT.IQGSHFANLLQASQANQLVIER.+3b10.light | 39,2 |
| 883,437 | 1132,538 | tr\|Q2RPT8\|Q2RPT8_RHORT.GQTGLSVAFDLPTQTGYDSDHVLAR.+3y10.light | 42,5 |
| 883,437 | 797,426 | tr\|Q2RPT8\|Q2RPT8_RHORT.GQTGLSVAFDLPTQTGYDSDHVLAR.+3y7.light | 42,5 |
| 883,437 | 595,367 | tr\|Q2RPT8\|Q2RPT8_RHORT.GQTGLSVAFDLPTQTGYDSDHVLAR.+3y5.light | 42,5 |
| 883,437 | 1003,484 | tr\|Q2RPT8\|Q2RPT8_RHORT.GQTGLSVAFDLPTQTGYDSDHVLAR.+3y18+2.light | 42,5 |
| 883,437 | 894,432 | tr\|Q2RPT8\|Q2RPT8_RHORT.GQTGLSVAFDLPTQTGYDSDHVLAR.+3y16+2.light | 42,5 |
| 883,437 | 780,376 | tr\|Q2RPT8\|Q2RPT8_RHORT.GQTGLSVAFDLPTQTGYDSDHVLAR.+3y14+2.light | 42,5 |
| 794,091 | 932,509 | tr\|Q2RPT8\|Q2RPT8_RHORT.VGVPISHLGDMQTLFEGIPLEK.+3y8.light | 38,3 |
| 794,091 | 785,44 | tr\|Q2RPT8\|Q2RPT8_RHORT.VGVPISHLGDMQTLFEGIPLEK.+3y7.light | 38,3 |
| 794,091 | 656,398 | tr\|Q2RPT8\|Q2RPT8_RHORT.VGVPISHLGDMQTLFEGIPLEK.+3y6.light | 38,3 |
| 794,091 | 486,292 | tr\|Q2RPT8\|Q2RPT8_RHORT.VGVPISHLGDMQTLFEGIPLEK.+3y4.light | 38,3 |
| 794,091 | 276,155 | tr\|Q2RPT8\|Q2RPT8_RHORT.VGVPISHLGDMQTLFEGIPLEK.+3y2.light | 38,3 |
| 794,091 | 1063,054 | tr\|Q2RPT8\|Q2RPT8_RHORT.VGVPISHLGDMQTLFEGIPLEK.+3y19+2.light | 38,3 |
| 677,819 | 964,51 | tr\|Q2RPT8\|Q2RPT8_RHORT.DSGMEVVYEGIR.+2y8.light | 35,9 |
| 677,819 | 835,467 | tr\|Q2RPT8\|Q2RPT8_RHORT.DSGMEVVYEGIR.+2y7.light | 35,9 |
| 677,819 | 736,399 | tr\|Q2RPT8\|Q2RPT8_RHORT.DSGMEVVYEGIR.+2y6.light | 35,9 |
| 677,819 | 637,33 | tr\|Q2RPT8\|Q2RPT8_RHORT.DSGMEVVYEGIR.+2y5.light | 35,9 |
| 677,819 | 345,224 | tr\|Q2RPT8\|Q2RPT8_RHORT.DSGMEVVYEGIR.+2y3.light | 35,9 |
| 677,819 | 260,088 | tr\|Q2RPT8\|Q2RPT8_RHORT.DSGMEVVYEGIR.+2b3.light | 35,9 |
| 711,388 | 1054,542 | tr\|Q2RPT8\|Q2RPT8_RHORT.AEGLADIPVIVGGIIPPEDEK.+3y10.light | 34,4 |
| 711,388 | 827,415 | tr\|Q2RPT8\|Q2RPT8_RHORT.AEGLADIPVIVGGIIPPEDEK.+3y7.light | 34,4 |
| 711,388 | 714,33 | tr\|Q2RPT8\|Q2RPT8_RHORT.AEGLADIPVIVGGIIPPEDEK.+3y6.light | 34,4 |
| 711,388 | 617,278 | tr\|Q2RPT8\|Q2RPT8_RHORT.AEGLADIPVIVGGIIPPEDEK.+3y5.light | 34,4 |
| 711,388 | 979,546 | tr\|Q2RPT8\|Q2RPT8_RHORT.AEGLADIPVIVGGIIPPEDEK.+3b10.light | 34,4 |
| 711,388 | 1192,657 | tr\|Q2RPT8\|Q2RPT8_RHORT.AEGLADIPVIVGGIIPPEDEK.+3b13.light | 34,4 |
| 442,29 | 770,488 | tr\|Q2RPT8\|Q2RPT8_RHORT.ILLAAGVAR.+2y8.light | 24,1 |
| 442,29 | 657,404 | tr\|Q2RPT8\|Q2RPT8_RHORT.ILLAAGVAR.+2y7.light | 24,1 |
| 442,29 | 544,32 | tr\|Q2RPT8\|Q2RPT8_RHORT.ILLAAGVAR.+2y6.light | 24,1 |
| 442,29 | 473,283 | tr\|Q2RPT8\|Q2RPT8_RHORT.ILLAAGVAR.+2y5.light | 24,1 |
| 442,29 | 402,246 | tr\|Q2RPT8\|Q2RPT8_RHORT.ILLAAGVAR.+2y4.light | 24,1 |
| 442,29 | 227,175 | tr\|Q2RPT8\|Q2RPT8_RHORT.ILLAAGVAR.+2b2.light | 24,1 |
